# Human C1q Regulates Influenza A Virus Infection and Inflammatory Response via Its Globular Domain

**DOI:** 10.3390/ijms23063045

**Published:** 2022-03-11

**Authors:** Praveen M. Varghese, Uday Kishore, Reena Rajkumari

**Affiliations:** 1 Biosciences, College of Health, Medicine and Life Sciences, Brunel University London, Uxbridge, London UB8 3PH, UK; praveenmathewsvarghese@gmail.com; 2 School of Biosciences and Technology, Vellore Institute of Technology, Vellore 632014, India

**Keywords:** innate immunity, complement, classical pathway, immune evasion, human C1q, RNA viruses, influenza A virus, cytokine storm

## Abstract

The Influenza A virus (IAV) is a severe respiratory pathogen. C1q is the first subcomponent of the complement system’s classical pathway. C1q is composed of 18 polypeptide chains. Each of these chains contains a collagen-like region located at the N terminus, and a C-terminal globular head region organized as a heterotrimeric structure (ghA, ghB and ghC). This study was aimed at investigating the complement activation-independent modulation by C1q and its individual recombinant globular heads against IAV infection. The interaction of C1q and its recombinant globular heads with IAV and its purified glycoproteins was examined using direct ELISA and far-Western blotting analysis. The effect of the complement proteins on IAV replication kinetics and immune modulation was assessed by qPCR. The IAV entry inhibitory properties of C1q and its recombinant globular heads were confirmed using cell binding and luciferase reporter assays. C1q bound IAV virions via HA, NA and M1 IAV proteins, and suppressed replication in H1N1, while promoting replication in H3N2-infected A549 cells. C1q treatment further triggered an anti-inflammatory response in H1N1 and pro-inflammatory response in H3N2-infected cells as evident from differential expression of TNF-α, NF-κB, IFN-α, IFN-β, IL-6, IL-12 and RANTES. Furthermore, C1q treatment was found to reduce luciferase reporter activity of MDCK cells transfected with H1N1 pseudotyped lentiviral particles, indicative of an entry inhibitory role of C1q against infectivity of IAV. These data appear to demonstrate the complement-independent subtype specific modulation of IAV infection by locally produced C1q.

## 1. Introduction

Influenza, also known as the flu, is an acute viral infection that affects the upper or lower respiratory tract. The disease is characterised by the decimation of the infected respiratory cells, damage to the respiratory epithelium, and immunological responses that cause pneumonia and general discomfort, such as body ache, coughing, sneezing, high fever, sore throat, rhinorrhoea, myalgia and fatigue [1]. Influenza viruses are negative-strand RNA viruses belonging to the Orthomyxoviridae family. They are classified into four genera: influenza A virus (IAV), influenza B virus (IBV), influenza C virus (ICV), and influenza D virus (IDC). The classification is based upon antigenic differences in two major structural proteins, the nucleoprotein (NP) and the matrix protein (M) [2]. IAV and IBV cause outbreaks and epidemics; the ICV causes mild infections and is detected in much fewer cases [3]. However, only the IAV has been attributed to causing pandemics that can have devastating consequences if not treated or prevented. They occur approximately three times every century when viral antigens present in avian circulating strains are acquired by human influenza strains [4].

IAVs are classified into subtypes according to the properties of their major membrane glycoproteins, hemagglutinin (HA) and neuraminidase (NA). Eighteen HA subtypes (H1 through H18) and 11 NA subtypes (N1 through N11) have been identified among IAV [5]. The most routinely circulating subtypes in humans include A(H1N1) and A(H3N2). This vast range of variabilities in antigens can be associated with antigenic drift (minor changes in amino acid sequence caused by frequent mutations and genetic rearrangement) and antigenic shift (substantial changes in residues caused by frequent mutations and genetic rearrangement) [6].

Both HA and NA are essential for the entry and exit of virus particles at the plasma membrane of infected cells [7]. The attachment of the virus to the α-2,6-linked or α-2,3-linked sialic acid in the human tracheal epithelial cells is mediated by HA [8], the primary viral antigen responsible for inducing a virus neutralising host immune response [9,10]. The HA molecule is synthesised as a trimer with two regions consisting of unique structures cleaved by serine proteases into HA1 and HA2 subunits. The splicing activity is essential for the infectivity of the virus as HA1 is the globular domain at the distal end that contains receptor binding and antigenic sites. The N-terminus of HA2 triggers the fusion of the virus envelope with an endosomal compartment in acidic conditions after entry [11,12]. NA also helps in the entry of the virus into the host cell by cleaving the sialic acids found in the mucus surrounding the respiratory tract. Without NA, the virion would be trapped in the mucus and will have no access to the host cell [13,14]. Its primary function is to break the α-ketosidic linkage between the terminal sialic (N-acetylneuraminic) acid and a sugar residue. The acidic environment in the endosomal compartment also triggers the IAV virion internal acidification through a 97-amino acid homo-tetramer ion channel, called M2 [15]. This internal acidification disrupts the internal protein–protein interactions between the viral ribonucleoprotein complexes (RNPs) and M1, releasing the viral RNPs into the host cytoplasm [16,17,18]. The released RNPs then undergo replication, protein synthesis, packaging, and then culminating into budding and release of new IAV virions.

The innate immune response against invading IAV is critical for reducing viral proliferation and invasion. Once an IAV infection begins, the conserved viral components are recognised by the host’s pattern recognition receptors (PRRs), such as retinoic acid-inducible gene-I protein (RIG-I) and toll-like receptors (TLRs) [19,20]. RIG-1 recognises the intracellular ssRNA and transcriptional intermediates [21]. TLR3, TLR7, and TLR8 are involved in sensing the IAV components in the cytoplasmic endosomes during the virus replication [22,23,24]. Additionally, NOD-like receptors, such as the NOD-like receptor family pyrin domain containing 3 (NLRP3) and NLR apoptosis inhibitory protein 5, are also known to become activated during an IAV infection [25]. The activation of RIG-1 by IAV PAMPs triggers conformational changes in the PRR, causing the exposure of its caspase activation and recruitment domains (CARDs) [26]. The dephosphorylation or ubiquitination by E3 ligases then modulates the CARDs [26]. This CARD-dependent association of RIG-1 and the MAVS triggers a cascade of downstream signals at the outer mitochondrial membrane, which activates multiple transcription factors such as nuclear factor kappa-light-chain-enhancer of activated B cells (NF–κB), interferon regulatory factor (IRF) 3 and 7 [27,28]. These transcription factors, in turn, trigger the expression of a multitude of cytokines. Similarly, the activation of specific TLRs is triggered by the recognition of particular IAV components. For example, TLR3 is known to recognise dsRNA in endosomes and unfamiliar RNA structures that are present in phagocytosed cells infected with IAVs [29,30]. TLR8 is another PRR involved in IAV recognition, which senses ssRNA in monocytes and macrophages, and triggers the production of IL-12 [31]. TLR7 recognises the IAV ssRNA in plasmacytoid dendritic cell (pDC) endosomes [32]. The adaptor protein myeloid differentiation factor 88 in pDCs then triggers the downstream signalling cascade of TLR7, resulting in the activation of either NF–κB or IRF7 to induce the expression of pro-inflammatory cytokines and type I IFNs, respectively [32]. NLPR3 is expressed in DCs, macrophages, neutrophils, monocytes and human pulmonary epithelial cells; it is another PRR that is involved in IAV detection [33,34]. Once NLPR3 recognises IAV PAMPs, it induces the expression of pro-IL-1β, pro-IL-18, and pro-caspase-1 [35]. IAV M2 ions channels then trigger the cleavage of the pro-IL-1β and pro-IL-18 and the activation of the NLPR3 inflammasome [36]. Finally, the accumulation of the PB1-F2 protein of IAV triggers the activation of the NLPR3 inflammasome, and via transcription factors, production of cytokines and chemokines [37].

The cytokines and chemokines released by the infected airway epithelial cells trigger the rapid recruitment of innate immune effector cells, such as NK cells, monocytes, and neutrophils [21]. NK cells are cytotoxic lymphocytes in the innate immune system that are critical for eliminating IAV infection. HA expression on the surface of infected cells serves as a recognition signal for NK cells, which then target and lyse the virus-infected cells [38,39]. The binding of HA to the natural cytotoxicity receptors (NCRs) of NK cells, NKp44 and NKp46, mediates this lysis [40]. During an IAV infection, DCs, which are specialised antigen-presenting cells linking the innate and adaptive immune responses, are also recruited. Conventional DCs (cDCs) move from the lungs to lymph nodes after infection with IAV [41]. Antigenic peptides processed from IAV by cDCs are presented to T lymphocytes in lymph nodes [42,43]. Through their MHC class I and MHC class II molecules, self-infected DCs induce virus-specific CD8^+^ cytotoxic T lymphocytes (CTL) and CD4^+^ T helper (Th) cells, respectively. B cell proliferation (and maturation) into antibody-producing plasma cells is aided by CD4^+^ T helper cell activation [44]. DCs can also have cytolytic activity and contribute to creating the BALT (bronchus-associated lymphoid tissue) [45]. The recruited alveolar macrophages are also crucial for restricting viral propagation. By phagocytosing IAV-infected cells, activated macrophages modulate the adaptive immune response against the virus and can also directly restrict viral propagation, thus disrupting the cycle of infection [46].

The complement system, a major humoral wing of the innate immunity, provides another essential barrier against IAV infection [47]. The complement system can be triggered via three ways: classical, alternative, and lectin, depending on the recognition subcomponents and ligands that activate it. C1q binds to IgG or IgM-containing immune complexes or other non-immunoglobulin targets to set off the classical pathway. The autoactivation of serine protease C1r, which then cleaves and activates another serine protease, C1s, is triggered when C1q binds to antibodies or pathogen surfaces [48]. This results in the formation of a C1-complex that consists of one C1q molecule and two C1r and C1s molecules. C4 and C2 are then cleaved by the C1 complex, yielding C4a, C4b, C2a, and C2b. The C4b2a complex, also known as the C3-convertase, is formed when the C4b and C2a link together. C3 is cleaved into C3a and C3b by the C3 convertase enzyme. C3b forms a complex with C4b2a (from both the classical and lectin pathways), transforming them into classical or lectin C5 convertase, respectively. C5 initiates the effector terminal phase of the complement system, which is the same for all three routes. The C5 convertases cleave C5 at the Arg751-Leu752 position on the α chain to form C5a and C5b [49]. The C5b serves as an epicentre for the assembly of membrane attack complex (MAC) [50]. C5b interacts with C6, and C5b6 complex is strengthened further by C7 binding. C7 interaction reveals transient lipid-binding sites, allowing the complex to attach to the cell membrane. This bond does not hurt the cell, but it marks it as a target for future attacks. The C5b-7 complex subsequently interacts with C8, resulting in the tetrameric complex C5b-8, which promotes the binding and polymerisation of 10 to 16 C9 molecules. The C5b-9 complex is inserted onto the microbial/infected cell surface at the end of the complement system’s terminal phase. This leads to the opsonisation and subsequent lysis of the microbe/infected cell.

The complement system has been shown to protect against IAV in various in vitro and in vivo studies [51,52,53,54,55]. In vivo studies using mouse models have established that during an IAV infection, the complement-mediated protection was a late phenomenon that occurred after day 5 post Infection (p.i.) [56]. It was also found that the complement-mediated protection is dependent on C3, C4 and Factor B, suggesting in vivo cross-talk between the classical and alternative pathways [56]. It has been suggested that the recognition of IAV by antibodies triggers the complement activation, leading to C3b deposition and direct neutralization of the virus. Additionally, the fragments C3d and C3a generated as a result of complement activation are found to enhance B cell responses and the effector CD4^+^ and CD8^+^ T cell responses, respectively [56]. The effector CD8^+^ T cells and antibodies then efficiently contain the virus [56].

In vitro models have been used to investigate the IAV neutralising efficacy of C1q. At the cell-binding stage, C1q was reported to improve HA-specific monoclonal antibody-mediated suppression of IAV attachment to host cells (>100-fold) [57]. C1q also enhances the activity of anti-HA antibodies [58]. The globular portion of C1q A chain interacts with M1, a conserved multifunctional protein of IAV [59]. The N-terminal region of the M1 protein is involved in the interaction between M1 and C1qA chain [59]. In vitro, the M1 protein inhibits haemolysis and prevents complement-mediated neutralisation of IAV by blocking C1q A chain interaction with heat-aggregated IgG [59]. Given a range of functions of C1q that do not involve classical pathway activation, we have examined if C1q on its own can have a protective role against IAV in a serum-free condition. This is relevant since monocytes, macrophages and DCs are capable of synthesising C1q at the site of infection and inflammation [60,61,62,63,64].

We show here that C1q can modulate IAV infectivity independent of the complement activation or antibody response in a subtype dependent manner. C1q acts as an entry inhibitor for the H1N1 subtype when challenged against A549 (lung epithelial) cells and promotes an acute pro-inflammatory cytokine response. However, C1q was found to promote viral entry and an anti-inflammatory cytokine response in the case of the H3N2 subtype.

## 2. Results

### 2.1. Immobilised Human C1q and Its Recombinant Globular Head Modules Bind Influenza A Virus Subtypes

**Figure 1:**
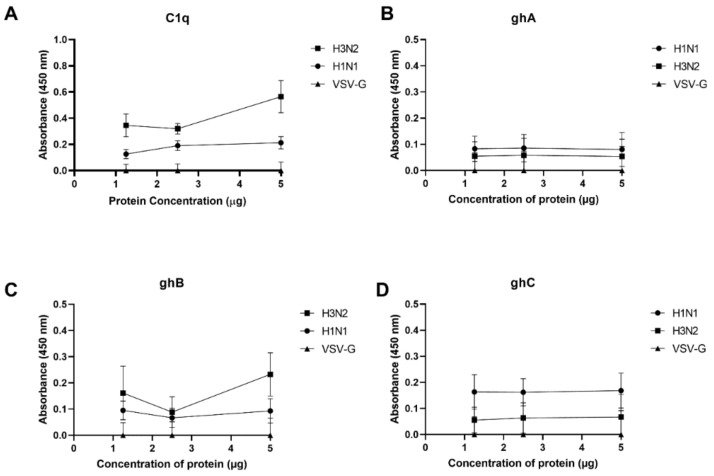
Interaction of H1N1 and H3N2 subtypes of influenza A virus (IAV) with C1q (**A**), ghA (**B**), ghB (**C**) and ghC (**D**). Decreasing concentrations (5, 2.5, and 1.25 μg) of human C1q and its recombinant globular head modules were coated overnight in a 96-microtiter well plate in carbonate/bicarbonate (CBC) buffer, pH 9.6 at 4 °C. Next, the wells were washed three times with PBS. Then, 20 µL of H1N1 or H3N2 virus (1.36 × 10^6^ pfu/mL) was added to corresponding wells and incubated at 37 °C for 2 h. After removing and washing off the unbound viruses, the wells were probed with primary antibodies (100 μL/well): monoclonal anti-influenza virus H1 or anti-influenza virus H3 (1:5000) antibodies, respectively. VSV-G pseudo-typed lentivirus was used as a negative control. The data were expressed as the mean of three independent experiments carried out in triplicate ± SD. The background was subtracted from all samples. In addition, the absorbance of Maltose Binding Protein (MBP) (5, 2.5, and 1.25 μg) was subtracted from the respective absorbance of the recombinant MBP tagged globular head modules (**B**–**D**).

**Figure 2:**
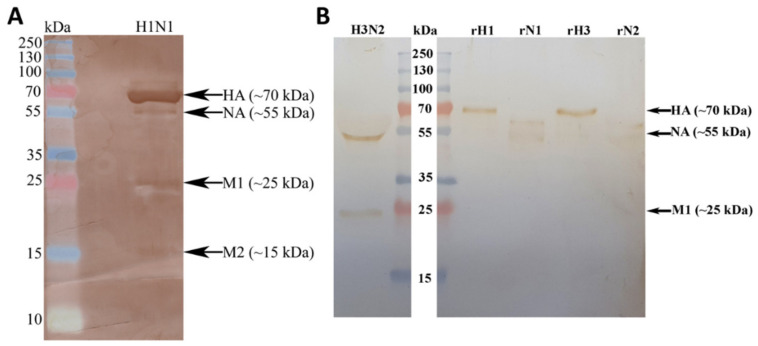
Far-Western blotting analysis to assess C1q binding to individual IAV proteins in the virus lysate, or purified H1N1 (**A**) and H3N2 (**B**). H1N1 and H3N2 virus lysates, or recombinant IAV glycoproteins (5 μg/mL) were separated using SDS-PAGE (12% *w*/*v*) under reducing conditions, and then transferred onto an activated PVDF membrane. Following blocking with PBS + 5% *w*/*v* BSA, the membrane was incubated with 20 μg/mL of C1q. After PBS washes, the membrane was probed with rabbit anti-human C1q antibody (1:1000). C1q bound M1 (~25 kDa), HA (~70 kDa) and NA (~55 kDa) of both IAV subtypes. C1q was also found to bind to the M2 protein of H1N1 alone.

ELISA was performed to assess the binding of C1q and its recombinant globular head modules to IAV subtypes (Figure 1). Immobilised C1q bound H1N1 and H3N2 at all concentrations tested (Figure 1A). Similarly, all three globular heads directly interacted with both IAV subtypes (Figure 1B–D). VSV-G pseudo-typed lentiviral particles were used as a negative control. Far-Western blotting was performed in order to determine the IAV proteins that interacted with human C1q (Figure 2). H1N1 and H3N2 lysates were separated on a 12% *w*/*v* SDS-PAGE gel and transferred to a PVDF membrane. The separated viral proteins were probed with 20 μg/mL C1q, and the interactions were detected using polyclonal antibodies against C1q. C1q was found to bind surface glycoproteins HA at ~70 kDa and NA at ~55 kDa, in addition to (M1 at ~25 kDa) of both IAV subtypes (Figure 2). C1q was also found to interact with matrix protein 2 (M2; ~15 kDa) in the case of the H1N1 subtype (Figure 2). Non-specific interaction of the anti-C1q polyclonal antibody with IAV proteins was ruled out by probing IAV lysates directly with the antibody (Figure S1).

### 2.2. Human C1q Modulates IAV Replication in A549 Lung Epithelial Cells

**Figure 3:**
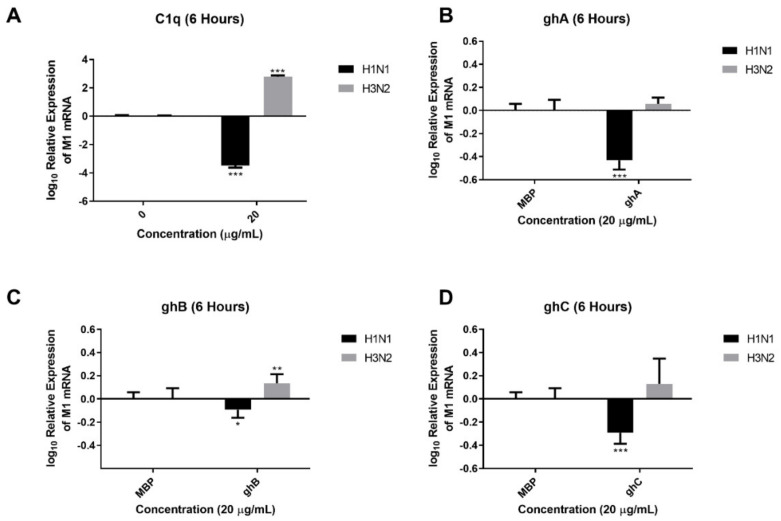
IAV pre–treatment with human C1q (**A**), ghA (**B**), ghB (**C**) or ghC (**D**) suppresses replication of H1N1-infected A549 cells while upregulating replication in H3N2. mRNA transcript levels of M1 expression of H1N1 and H3N2 IAV subtypes (IAV) (MOI 1) 6 h post-infection in A549 cells were measured. A549 cells were incubated with H1N1 or H3N2, pre–treated with or without human C1q or its recombinant globular head modules (20 μg/mL). Following cell lysis, RNA was extracted and converted into cDNA. M1 expression levels were measured via qRT–PCR using M1 primers to assess IAV replication; 18S was used as an endogenous control. Data are shown as the normalized mean of three independent experiments performed in triplicate ± SEM. C1q (**A**) was normalized to M1 levels of its control (cells + virus only), and the globular heads (**B**–**D**) were normalized to M1 levels of their control (Cells+ virus pre–treated with 20 μg/mL of MBP). Significance was determined using the two–way ANOVA test (* *p* < 0.05, ** *p* < 0.01, *** *p* < 0.001, (*n* = 3)).

The effect of C1q and its globular head modules on IAV infectivity and replication was assessed using an infection assay. H1N1 and H3N2 virions, pre-treated with C1q or its globular heads (20 µg/mL), exhibited differential expression of M1 mRNA levels 6 h post-infection in A549 cells (Figure 3). A549 cells infected with H1N1 or H3N2 virions alone were used as the control for the virions pre-treated with C1q. IAV virions pre-treated with MBP (20 µg/mL) were used as controls for the virions pre-treated with recombinant globular heads. Compared to their respective controls, viral M1 transcription of H1N1 virions was found to be significantly downregulated after treatment with C1q (~−3.5 log_10_) (Figure 3A), ghA (~−0.4 log_10_) (Figure 3B), ghB (~−0.3 log_10_) (Figure 3C), or ghC (~−0.1 log_10_) (Figure 3D). However, a significant upregulation in the viral M1 transcript levels was observed with H3N2 virions following treatment with either C1q (~3.0 log_10_) (Figure 3A), ghB (~−0.1 log_10_) (Figure 3C), or ghC (~−0.1 log_10_) (Figure 3D), compared to their respective controls. Treatment of H3N2 virions with ghA (~−0.0 log_10_) (Figure 3B) did not cause significant differences in M1 mRNA expression. These results suggested that C1q treatment differentially modulated IAV replication.

### 2.3. Human C1q Modulates IAV Entry in A549 Lung Epithelial Cells by Inhibiting IAV-Cell Interaction

**Figure 4:**
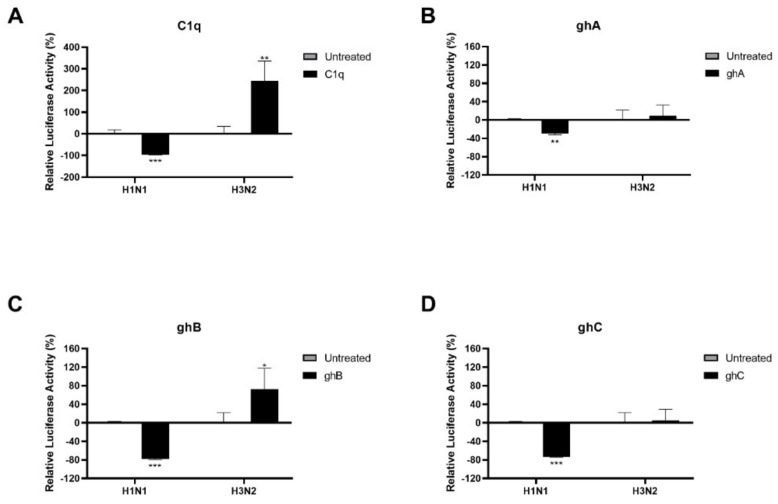
C1q, ghA, ghB, or ghC treatment modulates IAV entry in MDCK cells in a subtype–dependent manner. Matched H1N1 or unmatched H3N2 pseudo–typed lentiviral particles were pre–treated with C1q or its recombinant globular head modules (20 μg/mL). Luciferase reporter activity of MDCK cells transduced with either treated or untreated pseudo–typed lentiviral particles was measured to assess if the protein treatment affected the ability of the virus to enter the cells. The pre––treatment of the viral particle with immune proteins was found to inhibit viral entry in the case of H1N1 particles but to promote viral entry in H3N2 particles. C1q (**A**) was normalized to relative luminescence unit of its control (cells + virus only), and the globular head modules (**B**–**D**) were normalized to relative luminescence unit levels of their control (Cells+ virus pre–treated with 20 μg/mL of MBP). Data are shown as the normalized mean of three independent experiments performed in triplicate ± SEM. Significance was determined using the two–way ANOVA test (* *p* < 0.05, ** *p* < 0.01, *** *p* < 0.001, (*n* = 3).

HA and NA play a critical role in the recognition of sialic acid residues on host cells and subsequent viral entry. Thus, lentiviral pseudo-types were generated to study the effect of C1q and its globular head modules on the viral cell entry of H1N1 and H3N2 subtypes (Figure 4). Purified matched H1 + N1 and unmatched H3 + N2 pseudo-typed particles, pre-treated with C1q, ghA, ghB, or ghC (20 μg/mL), were used to infect MDCK cells. MDCK cells transduced with H1 + N1 or H3 + N2 virions alone were used as the control for the pseudo-typed particles pre-treated with C1q. Similarly, pseudo-typed particles pre-treated with MBP (20 µg/mL) were used as control for the pseudo-typed particles. Significantly reduced luciferase activity was recorded in H1 + N1 pseudotyped particles pre-treated with C1q (~−97%; −1.46 log_10_) (Figure 4A), ghA (~−30%; −0.15 log_10_) (Figure 4B), ghB (~−78%; −0.67 log_10_) (Figure 4C), or ghC (~−74%; −0.58 log_10_) (Figure 4D) when compared to the control. However, an opposite effect was observed with H3 + N2 pseudotyped particles pre-treated with C1q (~244%; 0.54 log_10_) (Figure 4A), or ghB (~73%; 0.24 log_10_) (Figure 4C) when compared to their control. No significant differences in luciferase activity between the controls and the H3 + N2 pseudotyped particles treated with ghA (~9%; 0.04 log_10_) (Figure 4B) or ghC (~5%; 0.02 log_10_) (Figure 4D) was observed. Hence, C1q protein via ghB, ghC and, to a lesser extent, ghA acts as an entry inhibitor for H1N1, but as a strong facilitator of infection for H3N2, most likely via ghB.

**Figure 5:**
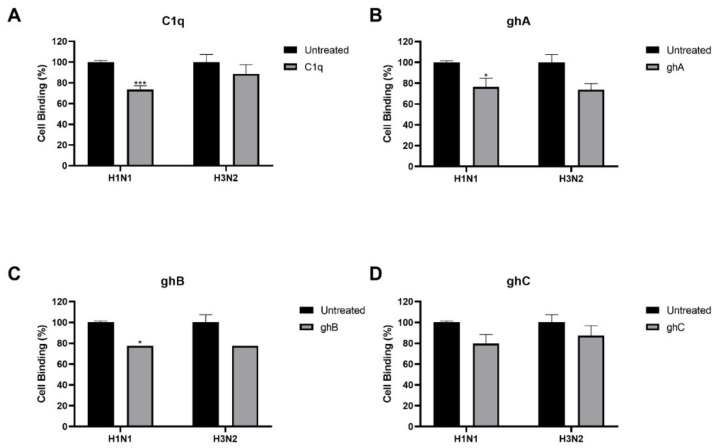
Pre-treatment of IAV with human C1q restricts H1N1 viral particles binding to the cell surface. A549 cells (1 × 10^5^ cells/mL) were infected with H1N1 or H3N2 viral particles pre-incubated with or without human C1q (**A**), ghA (**B**), ghB (**C**) or ghC (**D**) (20 µg/mL). Then, 2 h post-infection, unbound protein and viral particles were removed, and the wells were fixed with 1% *v*/*v* paraformaldehyde for 1 min. The wells were probed with the corresponding primary antibodies: monoclonal anti-influenza virus H1 or polyclonal anti-influenza virus H3 antibodies (1:5000). Data are shown as the normalized mean of three independent experiments performed in triplicate ± SEM. Data were normalized to the absorbance of its untreated control (cells + virus only). Significance was determined using the two-way ANOVA test (* *p* < 0.05, *** *p* < 0.001, (*n* = 3)).

The entry inhibitory activity exhibited by C1q against the H1N1 subtype suggested that C1q blocked IAV at an early stage of the infection cycle. Since C1q and its globular head modules were found to interact with HA and NA, a cell-binding assay was performed to confirm if C1q binding to the IAV proteins inhibited the interaction between IAV and its cell surface receptors on A549 cells (Figure 5). Treatment of H1N1 virions with C1q reduced IAV binding to A549 cell surface by ~30 % (Figure 5A). Similarly, treatment with ghA and ghB reduced H1N1-A549 binding by ~25% (Figure 5B,C). However, no significant difference in cell binding was observed when H1N1 virions were treated with ghC, or when H3N2 virions were treated with C1q or any of its recombinant globular head moduless (Figure 5A–D). Taken together, C1q appeared to reduce H1N1 infections by preventing the binding of H1N1 viral particles to the cell surface, and hence, inhibiting subsequent viral entry and replication.

### 2.4. IAV Infection Induced NF–κB Activation Is Modulated by Human C1q

**Figure 6:**
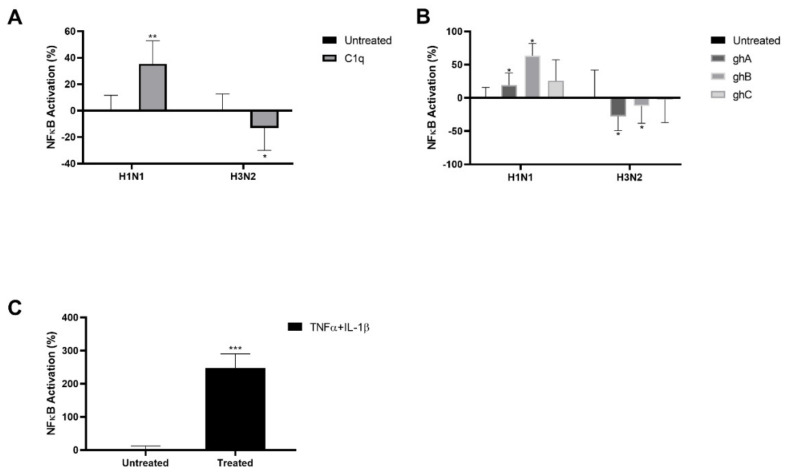
C1q treatment triggers NF-κB activation during H1N1 infection. NF–κB activation was measured via luciferase reporter assay in A549–NF–κB–Luc cells following challenge with C1q (**A**), globular-head- or MBP- (**B**) treated H1N1 or H3N2 subtypes. The relative NF-kB activity of C1q treated IAV challenged A549–NF–κB–Luc was calculated by using untreated sample (cells + virus only) as the baseline. The NF–κB activity of ghA-, ghB- or ghC-treated IAV-challenged A549–NF–κB–Luc cells only was calculated by using MBP-treated IAV-challenged A549–NF–κB–Luc the baseline. Cells treated with TNF-α and IL-β were used as a positive control for NF–κB activation (**C**). Data shown as the relative mean of three independent experiments ± SEM. Significance was determined using the two-way ANOVA test (* *p* < 0.05, ** *p* < 0.01, *** *p* < 0.001, (*n* = 3)).

The levels of the NF–κB activation in A549 cells, challenged with C1q, ghA, ghB, ghC or MBP-treated H1N1 or H3N2, was assessed by the use of NF–κB reporter assay (Figure 6). A549–NF–κB–Luc cells challenged with C1q-treated H1N1 virions reported ~35% increase in NF–κB Activation, while the cells challenged with the treated H3N2 virions exhibited ~13% reduction in NF–κB activity, compared to their respective untreated controls (Figure 6A). Compared to A549–NF–κB–Luc cells challenged with MBP-treated H1N1, ghA, ghB or ghC were found to upregulate NF–κB activity in cells challenged with the respective globular heads-treated H1N1 by ~19%, 64% and 2% respectively, (Figure 6B). However, NF–κB activation was downregulated in A549–NF–κB–Luc cells challenged with ghA- (~−28%), ghB- (~12%), or ghC- (~−2%) treated H3N2, compared to the MBP-treated H3N2 controls (Figure 6B). A549–NF–κB–Luc cells treated with TNFα and IL-1β were used as positive controls for the activation of NF–κB (Figure 6C).

### 2.5. Modulation of Immune Responses by C1q on A549 Cells Infected with IAV Subtypes

The study used qPCR to assess the modulation of the immune response by C1q and its globular heads on A549 cells challenged with H1N1 and H3N2 subtypes (Figure S3 and Table 1). Six hours post-infection, TNF-α mRNA levels were upregulated in A549 cells challenged with C1q-treated H1N1 (~0.15 log_10_) or H3N2 (~0.4 log_10_). In contrast, at the earlier 2 h time point, a downregulation (~−0.4 log_10_) in TNF-α mRNA level was observed in the A549 cells challenged with C1q-treated H3N2 virus. Levels of IL-12 mRNA at 2 h in the case of A549 cells challenged with C1q treated H1N1 or H3N2 were not detectable. At 6 h in C1q-treated H1N1 challenged cells, IL-12 levels were slightly upregulated (~0.5 log_10_). In cells challenged with C1q-treated H3N2, the levels were highly upregulated (~1.5 log_10_). However, no change in the mRNA expression levels of IL-6 at 2 h in A549 cells challenged with C1q-treated H3N2 virus was observed. At 6 h in C1q-treated H1N1 or H3N2 infected cells, IL-6 mRNA levels (~−0.3 log_10_) were downregulated. C1q downregulated IFN-α in both H1N1/H3N2-infected cells at 6 h. C1q-treated H1N1-challenged A549 cells showed lower mRNA levels of IFN-α at 6 h (~−0.5 log_10_). In contrast, cells challenged with C1q-treated H3N2 exhibited a greater reduction (~−2 log_10_) in the expression levels of IFN-α mRNA at 2 h time-point. The IFN-α mRNA levels returned to baseline 6 h post-infection. In the case of NF-κB levels, C1q treatment induced differential effects in both H1N1- and H3N2-infected cells. NF–κB mRNA levels at 6 h were slightly upregulated (~0.1 log_10_) in C1q-treated H1N1 challenged cells. A slight decrease (~−0.2 log10) in its levels was observed in cells challenged with C1q-treated H3N2 at 2 and 6 h post-infection. No cytokine expression was detected in C1q-treated H1N1 viruses 2 h post-infection.

A similar analysis was conducted using ghA-, ghB-, ghC-treated H1N1 challenged A549 cells. In H1N1-infected cells, a higher expression of TNF-α mRNA was observed 2 h post-infection [ghA: (~2.4 log_10_); ghB: (~3.20 log_10_); ghC: (~2.82 log_10_)], with expression levels decreasing to a greater than control level at the 6 h time-point for ghA- (0.63 log_10_) or ghB- (~0.27 log_10_) treated samples. Similarly, NF-κB expression was also upregulated after treatment with globular heads at both time points tested; it was higher 2 h post-infection when treated with ghB (~0.33 log_10_) or ghC (~0.47 log_10_), with expression levels slightly reduced when tested at 6 h post-infection. However, even at the 6 h time point, the NF-κB mRNA expression was upregulated in the samples treated with ghA (~0.14 log_10_) or ghB (~0.15 log_10_). Despite the TNF-α and NF-κB mRNA expression being upregulated, IFN-α mRNA levels were found to be downregulated at both the 2 and 6 h time-point. Treatment with ghB decreased (~−0.45 log_10_) IFN-α mRNA levels 2 h post-infection. The levels returned to slightly lower than controls 6 h post-infection (~−0.10 log_10_). However, treatment with ghC lowered IFN-α mRNA levels by almost two-fold 6 h post-infection (~−0.55 log_10_), compared to levels observed 2 h post-infection (~−0.25 log_10_). ghA treatment did not have any effect on IFN-α mRNA levels. The IL-6 mRNA expression levels were found to be upregulated irrespective of the globular head modules used. No expression of IL-6 mRNA was detected 2 h post-infection in ghA- and ghC-treated samples at the 6 h timepoint. However, treatment with ghA (~0.50 log_10_) or ghC (~1.84 log_10_) upregulated the IL-6 mRNA expression at the other time point. ghB-treated samples showed a higher expression of IL-6 mRNA at the 2 h time point (~1.98 log_10_) than at the 6 h (~0.18 log_10_). Treatment with the globular heads modulated IL-12 mRNA expression levels differentially. Treatment with either ghA (~−0.93 log_10_) or ghB (~−0.14 log_10_) downregulated IL-12 mRNA levels 2 h post-infection. Nevertheless, mRNA levels increased at 6 h in both ghB (~0.34 log_10_) and ghA (~1.17 log_10_). Treatment with ghC was found to cause a slight upregulation in IL-12 mRNA levels at both 2 h (~0.19 log_10_) and 6 h (~0.23 log_10_) time-points. Treatment with either ghA (~−0.39 log_10_) or ghB (~−0.40 log_10_) downregulated RANTES mRNA levels at the 2 h time-point. However, 6 h post-infection, the expression level of RANTES mRNA increased in both ghA- (~0.17 log_10_) and ghB- (~0.23 log_10_) treated samples. No RANTES mRNA was detected 2 h post-infection after treatment with ghC. However, the expression of RANTES mRNA was downregulated (~−0.52 log_10_) at the 6 h time-point followingr ghC treatment.

In the case of A549 cells infected with H3N2 in the presence of ghA, ghB or ghC modules, TNF-α was downregulated 2 h post-infection by ghA (~−0.29 log_10_) and ghC (~−0.11 log_10_). However, cells challenged with ghB treated virions exhibited an upregulation in TNF-α expression (~0.31 log_10_)−2 h post infection. No changes were observed in TNF-α mRNA expression 6 h post-infection after treatment with ghA. Although ghB (~−0.25 log_10_) treatment downregulated the expression, ghC (~0.39 log_10_) treatment upregulated TNF-α mRNA expression 6 h post-infection. Unlike NF-κB mRNA expression observed in the cells infected with the globular-head-treated H1N1, NF-κB mRNA expression was downregulated 6 h post infection with H3N2 pre-treated with ghA (~−0.42 log_10_) or ghB (~−0.21 log_10_). Nevertheless, no NF-κB mRNA modulation was observed in cells at 6 h due to ghC-treated H3N2 infection. In addition, 2 h post infection, NF-κB expression was upregulated by globular head modules [ghA: (~0.77 log_10_); ghB: (~0.60 log_10_); ghC: (~0.65 log_10_)]. Similarly, the IFN-α was downregulated in the cells infected with H3N2 pre-treated with ghA, ghB or ghC at both the time-points tested. Both ghA and ghC treatment increased the downregulation of IFN-α mRNA from 2 h [ghA: (~−0.96 log_10_); ghC: (~−0.51 log_10_)] to 6 h [ghA: (~−1.88 log_10_); ghC: (~−1.75 log_10_)). However, ghB was found to have a greater downregulation at 2 h (~−2.15 log_10_) compared to 6 h (~−0.23 log_10_). IL-6 mRNA expression was found to be upregulated after ghA (~0.30 log_10_) or ghB (~0.29 log_10_) treatment 2 h. No difference in expression was observed at the same time point after ghC treatment. At 6 h time-point, treatment with ghA (~−0.83 log_10_) or ghB (~−0.69 log_10_) showed a downregulation of IL-6 mRNA expression, while ghC (~0.25 log_10_) treatment caused an upregulation in IL-6 expression. No difference in IL-12 expression was observed 2 h post infection after treatment with the globular head modules. At the 6 h time-point, treatment with ghA (~−0.39 log_10_) or ghC (~−0.24 log_10_) downregulated IL-12 mRNA expression, but ghB (~0.71 log_10_) treatment upregulated it. RANTES mRNA expression was downregulated at the 6 h time-point after treatment with ghA (~−0.74 log_10_), ghB (~−0.29 log_10_) or ghC (~−1.36 log_10_). Although 2 h after the infection, ghA (~1.4 log_10_) or ghC (~0.35 log_10_) treatment increased RANTES mRNA expression, ghB (~−0.89 log_10_) treatment downregulated the expression of RANTES mRNA.

In summary, these results suggest a pro-inflammatory response by C1q and its globular head modules during H1N1 infection and an anti-inflammatory response during H3N2 infection.

## 3. Discussion

This study shows that human C1q, the recognition subcomponent of the classical pathway, significantly reduces the entry and subsequent replication of the H1N1 subtype of the IAV while promoting the entry of the H3N2 subtype. C1q was found to accomplish this subtype-specific modulation of IAV entry without the need for the other downstream components of the complement system or antibodies. The principal mechanism behind the complement independent inhibition of H1N1 entry may be an increase in steric inhibition due to deposition of the large C1q molecule onto the IAV via HA/NA-C1q interaction, and masking of viral receptor binding sites.

C1q is a 460 kDa protein that is made up of 18 polypeptide chains (6A chains, 6B chains, and 6C chains), each of which has a short N-terminal stem, a triple-helical collagen region, and a globular (gC1q) domain at the C-terminus [65,66]. The gC1q domain is known to bind to the antigen-complexed or -aggregated C_H_3 domain of IgM and the C_H_2 domains of certain IgG isotypes [58]. C1q can also interact directly with several viruses via its gC1q domain. C1q is known to bind envelope glycoproteins of several viruses, including gp41 and gp120 of HIV-1, p15E of murine leukaemia virus (MuLV), and gp21 of human T lymphotropic virus (HTLV)-1 [67,68,69,70]. C1q, its gC1q domain, and gC1q receptor, gC1qR, interact with gp41 of HIV-1 [71]. Furthermore, gC1qR has also been found to interact with a variety of other viral ligands, including HCV core protein, rubella viral capsid protein, adenovirus core protein V, and Epstein–Barr virus EBNA-1 [72,73,74,75]. Additionally, C1q binds the Chandipura virus directly with no effect on the virus’ infectivity [76]; however, viral neutralisation needs C1q-reconstituted serum [76].

In this study, C1q and the recombinant globular head modules were found to bind both H1N1 and H3N2 subtypes of IAV directly (in an antibody-independent manner). C1q specifically bound IAV envelope proteins, HA and NA. C1q bound M1 of both H1N1 and H3N2. However, C1q also bound the M2 protein of H1N1. Because the M1 protein is found beneath the lipid layer, the binding most likely occurs with the abundant non-packaged M1/M2 released from necrotic cells during late stages of infection to assist in viral survival [59].

The effect of C1q and its three recombinant globular head modules (ghA, ghB and ghC) on A549 cell viability was initially tested using the MTT assay to ensure that all effects observed were exclusively due to C1q and its recombinant globular head modules interacting with viral proteins. C1q or recombinant globular-head-treated A549 cells did not exhibit a substantially altered cell viability profile at a concentration of 20 µg/mL over 24 h incubation (Supplementary Figure S2). However, a decrease in cell viability was observed in A549 cells treated with a concentration of 40 µg/mL of C1q or ghC.

Using M1 mRNA expression levels, the ability of C1q and its recombinant globular head modules to modify viral replication in A549 cells was assessed. C1q modulated IAV replication in a strain-dependent manner. A549 cells challenged with C1q-treated H1N1 virions showed lower viral M1 expression compared to their controls. However, higher levels of viral M1 expression was observed in C1q-treated H3N2 virions-challenged A549 cells. This strain-dependent inhibitory effect of C1q may be explained by the varied effects of the C1q’s globular heads on IAV replication. Compared to the MBP-treated controls, all three heads were found to significantly reduce M1 levels in A549 cells challenged with H1N1 virions. However, only ghB-treated H3N2 virions significantly affected M1 expression when compared to its respective control. Thus, likely differences in C1q binding sites on HA or NA may arise from the local variation on H1 and H3 or N1 and N2.

The binding of C1q to HA and NA could block their interaction with their cell surface receptors, the sialic acids. This was confirmed with a cell-binding assay, which revealed a 30% decrease in H1N1-A549 binding after the virus was treated with C1q, and a 25% decrease after the virions were treated with ghA or ghB. No difference in binding was observed with H3N2 virions treated with C1q or the globular heads modules. Hence, C1q most likely controls viral replication levels by influencing viral entry into the cells. Second-generation matched and unmatched lentiviral vectors pseudo-typed for H1 + N1 and H3 + N2, respectively, were used to verify this arguement. Since the luciferase reporter gene containing pseudo-typed IAV particles are restricted to only one replicative cycle, this strategy was chosen as a safe surrogate model to simulate the structure and surfaces of IAV, and to demonstrate that C1q functions as an entry inhibitor. The assay revealed a four-fold reduction in luciferase activity in MDCK cells transduced with H1N1 pseudo-typed particles treated with C1q. However, in MDCK cells transduced with C1q treated H3N2 pseudo-typed particles, a 0.5-fold increase in luciferase activity was observed. Furthermore, all the three chains of C1q were found to significantly inhibit H1N1 entry, while only ghB was found to promote the entry of the H3N2 subtype significantly. Hence, consistent with the previous M1 expression data, this entry inhibition assay further confirms that C1q inhibits H1N1 entry into the host cells while having minimal restrictive efficacy against H3N2.

These results are in accordance with the previously reported data using the other complement regulators. The classical and alternative pathways regulators, C4BP and Factor H, respectively, were also found to modulate IAV infection in a subtype-specific manner, similar to C1q being reported here [47,77]. After treatment of H1N1 particles with factor H, Vaccinia virus complement control protein (VCP), or C4BP, A549 cells showed a decrease in M1 expression. However, A549 cells challenged with H3N2 virions treated similarly showed an increase in M1 expression [47,77]. Hence, the treatment with these proteins resulted in the restriction of IAV replication for the H1N1 subtype while promoting replication for H3N2 [47,77]. The ability of Factor H, VCP or C4BP to influence IAV entry in a subtype-dependent manner was also established using lentiviral vectors pseudo-typed for H1 + N1 and H3 + N2 [47,77]. The subtype-dependent entry modulation was found to occur via interactions with HA, NA, and M1, as seen in the case of C1q [47,77]. Thus, there seems a possible common mechanism that the complement effector and regulatory proteins use to modulate the IAV infection in a complement activation-independent manner.

IAV targets lung epithelial cells, and offspring viral particles grow after initial exposure to infect other cells, including alveolar macrophages [78]. The induction of pro-inflammatory cytokines and chemokines [78,79] to high levels results in a catastrophic cytokine storm. NF–κB is a critical regulator of innate immune surveillance against viral infection [80] because of its ability to induce the expression of a range of cytokines and chemokines [81,82,83]. Furthermore, the activation of the PRR triggers the activation of transcription factors such as, NF–κB, IRF3, and IRF7. Once activated, these transcription factors get translocated into the nucleus and trigger the transcription of IFNs (IFN-α and IFN-β, and type III IFNs) and pro-inflammatory cytokines (TNF, IL-6, IL-1β) [21]. The IFN-α, IFN-β and IFN-λs interact with IFN-α/β receptors (IFNAR) and IFNL receptors (IFNLR), respectively [84]. This interaction then triggers the activation of the Janus Kinase Signal Transducer and Activator of Transcription (JAK-STAT) signalling pathway, leading to the transcription of numerous IFN-stimulated genes (ISGs) [21]. Different stages of the IAV life cycle are targeted by these ISGs [84]. Several ISGs, such as the Mx family, interferon-induced transmembrane protein family (IFITMs), cholesterol 25-hydroxylase (CH25H), and TRIM proteins, have been shown to inhibit viral entry into host cells [21,84]. Hence, in this study, NF–κB activation was assessed using a NF–κB reporter assay. C1q and its recombinant globular head modules were found to promote NF–κB activity in A549 cells challenged with H1N1, while reducing NF–κB activity in H3N2 challenged cells. This variation can be attributed to the varying levels of IAV proteins, specifically NS1 protein, which is known to inhibit NF-κB activation. The NS1 levels in the C1q treated H1N1 challenged A549 cells may be sub-optimum allowing NF-κB activation to proceed and helping the host in viral clearance. In case of H3N2, the increased concentration of NS1 protein may be suppressing NF-κB activation and promoting viral survival and replication.

IAV infection induces production of higher levels of IL-6, TNF-α, IFNs, IL-1β, RANTES, IL-8, MIP1β, and MCP1 [85,86,87,88]. Furthermore, TNF-α, IL-12, and IFNs bring about immune infiltration. Additionally, IL-12 has been linked to the reduction of early viral replication during an IAV infection. The viral titre is much higher in cells treated with an anti-IFN-α/β receptor antibody, but not in dominant-negative IκBα expressing cells, suggesting that IAV infection can induce IFN expression via NF–κB [80]. Overproduction of IFN during the early stages of IAV infection can cause irreparable lung damage in H5N1-infected mice, yet IFN signalling is considered necessary in preventing H5N1 spread [89,90]. TNFs are important soluble factors in the cytokine storm; H5N1-infected mice lacking TNF receptors and H5N1-infected mice were given anti-TNF-α antibodies and had the same survival rate as the healthy controls [91]. Furthermore, during influenza infection, the host produces the pro-inflammatory IL-6 and IL-1. In the early stages of IAV infection, IL-1 expression is observed, followed by a rise in IL-6 expression [92]. Infected animals lacking the IL-6 receptor had a poor prognosis, implying that the IL-6 pathway is protective in the cytokine storm [92]. As a result, an imbalanced cytokine storm can disrupt the vascular barrier, resulting in tissue oedema, capillary leakage, multiple organ failure, and death [92]. When it comes to cytokine storm initiated by influenza strains, multiple mechanisms have been identified. People infected with IAV have higher amounts of pro-inflammatory cytokines such TNF- α, IL-6, and IFN- α in their blood [93]. IL-6 and TNF-α play a crucial role in viral-mediated respiratory disorders such as acute respiratory distress syndrome (ARDS) and acute lung injury [94]. Alveolar macrophages, the primary pulmonary phagocytic cells that release large amounts of IL-6 and TNF-α, are activated during IAV infection [95]. IAV-infected macrophages also produce chemokines such RANTES and monocyte chemotactic protein-1 (MCP-1). This increases the synthesis of cytokines (e.g., TNF- α, IL-6, RANTES, and IL-8) linked to IAV pathogenesis [96]. Influenza-induced RANTES, IL-1β, IL-6, and TNF-α induce pro-inflammatory Th1 immune responses in the infected host [96]. Dysregulation of cytokine and chemokine levels contributes to increased tissue injury and modulation of viral clearance.

To study the modulation of these cytokines by C1q and its globular head modules, we performed qPCR on A549 cells challenged with H1N1 or H3N2 that were pre-treated with C1q, ghA, ghB or ghC. The downregulation of TNF-α, NF–κB, IFN-α, IL-6, IL-12 and RANTES observed in treated H3N2 cells suggests that C1q treatment induces an anti-inflammatory state in A549 cells during the early stages of infection. The anti-inflammatory effect, while protecting from a cytokine storm, may be detrimental in the case of H3N2, as it may help in viral replication and prevent viral clearance. However, the expression profile of TNF-α, IFN-α, IL-6, IL-12 and RANTES 6 h post-infection was similar for both C1q-treated H1N1 and H3N2 subtypes. In the case of ghA-treated IAV, a distinct profile was observed among both the subtypes. The cytokine profile suggests that although ghA was not found to play a critical role in H3N2 entry or replication, it plays a crucial role in inducing an antiviral state that helps in efficient replication and viral dissemination. A549 cells infected with ghB-treated IAVs almost had the same cytokine profile among the two subtypes. Nevertheless, the downregulation of TNF-α, NF-κB, IL-6 and RANTES in the case of later stages of H3N2, compared to H1N1, suggests that ghB treatment at the later stages of H3N2 infection curbs the antiviral response and promotes viral replication.

Although these in vitro observations help establish the antiviral role of C1q against IAV infection using A549 cells, it would be worthwhile to replicate these outcomes in humanised murine models to mimic the microenvironment of the respiratory system and elucidate the subtype-dependent entry modulating mechanism. It would also be crucial to identify the residues involved in C1q-IAV interaction to help develop a specific C1q-derived HA/NA inhibitor to restrict early stages of IAV infection. Furthermore, it would be interesting to study the effects of C1q and its globular head modules in conjunction with established HA and NA inhibitors. Once the toxicological profile of the recombinant globular heads of C1q has been established in animal models, human infection challenge studies to investigate systemic and local immunity to IAV infections would provide a unique opportunity to investigate mechanisms of protection and disease severity in humans.

## 4. Materials and Methods

### 4.1. Purification of Human C1q

Human C1q was purified from freshly frozen human plasma [97]. Briefly, lipids were removed from human plasma by centrifugation at 5000× *g* for 10 min and Whatman filter paper. The plasma was then incubated with IgG- Sepharose for 2 h at 20 °C. The C1q bound beads were then washed with the wash buffer (10 mM HEPES, 140 mM, NaCl, 0.5 mM EDTA, pH 7.0). C1q was eluted using elution buffer (100 mM CAPS, 1 M NaCl, 0.5 mM EDTA, pH 11) and passed through a Hi-Trap Protein G column to remove residual IgG. This was followed by dialysis against 0.1 M HEPES buffer, pH 7.5.

### 4.2. Production of Recombinant Globular Heads of Human C1q

The recombinant globular head regions of human C1q A (ghA), B (ghB) and C (ghC) chains were expressed as a fusion to *Escherichia coli* maltose-binding protein (MBP) in *E. coli* BL21 cells and affinity-purified on an amylose resin column [71]. Briefly, the expression constructs were transformed into *E. coli* BL21 cells, and induced using 0.4 mM IPTG at OD_600_ = 0.6. Post induction, the cells were grown for a further 3 h at 37 °C. The cells were then centrifuged and lysed using lysis buffer (20 mM Tris–HCl, pH 8.0, 0.5 M NaCl, 0.1% *v*/*v* Tween 20, 1 mM EGTA pH 7.5, 1 mM EDTA pH 7.5, 5% *v*/*v* glycerol, 100 μg/mL lysozyme, 0.5 mM PMSF) for 30 min at 4 °C and sonicated. The lysate was then centrifuged at 15,000× *g* for 30 min. and the supernatant was diluted in buffer 1 (20 mM Tris–HCl, pH 8.0, 100 mM NaCl, 1 mM EDTA pH 7.5, and 5% *v*/*v* glycerol) and 0.1% *v*/*v* Tween 20. This was then passed through an amylose resin column and washed with buffer 1 with 0.2% *v*/*v* Tween 20 and then with only buffer 1. The globular head modules were eluted using buffer 1 containing 10 mM maltose. The eluted globular head modules were passed through Pierce™ High Capacity Endotoxin Removal Resin (Qiagen, Germantown, MD, USA) to remove endotoxin contamination.

### 4.3. Preparation of Viral Stocks and Titration

A/England/195/2009 (H1N1) and A/HK/99 (H3N2) subtypes of IAV were used in this study. In a 175 cm^2^ flask (Fisher Scientific), Madin Darby Canine Kidney (MDCK) (ATCC^®^ CCL-34™) cells were grown to 70% confluence in growth medium [DMEMF/12 with GlutaMAX™ Supplement (Gibco, Waltham, MA, USA) + 10% *v*/*v* Heat inactivated Foetal Bovine Serum (FBS) (Gibco) + 1% *v*/*v* Penicillin–Streptomycin (PS) (Gibco)] at 37 °C under 5% *v*/*v* CO_2_. The cells were washed with PBS (Fisher Bioreagents) twice to remove any traces of the growth medium. H1N1 and H3N2 IAV subtypes, diluted in serum-free DMEM, were added to the MDCK cells and allowed to adsorb on the cell surface for 1 h at 37 °C. Unbound viruses were washed away using PBS. The cells were further incubated for another 72 h at 37 °C in infection medium (DMEM + 1% PS + 0.3% *w*/*v* bovine serum albumin (BSA) + 1 µg/mL of l-1-Tosylamide-2-phenylethyl chloromethyl ketone (TPCK)-Trypsin) (Sigma-Aldrich, Burlington, MA, USA). Post incubation, the supernatant containing the viral particles was harvested by centrifugation at 3000× *g* at 4 °C for 15 min. The titre of the virus was determined to be 1 × 10^6^ PFU/mL by TCID_50_ using the Reed–Muench method [98].

### 4.4. Direct Binding ELISA

Direct interaction of IAV subtypes with human C1q and recombinant globular head modules was evaluated using ELISA by coating a varied concentration of C1q, recombinant globular head modules or MBP (5, 2.5 or 1.25 µg/well) in carbonate–bicarbonate (CBC) buffer, pH 9.6 (Sigma) at 4 °C overnight. Unbound proteins were removed by washing the wells three times with PBS and blocked using 2% *w*/*v* BSA in PBS (Fisher Scientific, Waltham, MA, USA) for 2 h at 37 °C. The wells were then washed three times with PBS, and 1000 PFU/mL of purified H1N1 and H3N2 were added to the wells to test the binding of the virus to immobilised C1q or recombinant globular heads. The plate was incubated for 2 h at 37 °C and then washed with PBST to remove any unbound viruses. VSV-G pseudo-typed lentivirus was used as a negative control. The binding was detected using respective primary antibodies (1:5000): monoclonal anti-influenza virus H1 HA, A/California/04/2009 (H1N1) pdm09, Clone 5C12 (produced in vitro) (BEI-Resources, NIAID, NIH, Manassas, VA, USA), polyclonal anti-influenza virus H3 HA, A/Hong Kong/1/1968 (H3N2) (antiserum, Goat, NR-3118) (BEI-Resources, NIAID, NIH, Manassas, VA, USA), polyclonal anti-VSV-G (1 mg/mL of purified IgG; Imperial College London). After 3 washes with PBST Buffer (PBS + 0.05% Tween 20) (Fisher Scientific), the binding was detected using respective secondary antibodies (1:5000); goat anti-mouse IgG HRP or goat-anti-rabbit IgG HRP. The colour was developed by adding 3,3′,5,5′-Tetramethylbenzidine (TMB) substrate (50 µL/well). The reaction was stopped using 1 M H_2_SO_4_ (50 µL/well), and absorbance was read at 450 nm using a Biorad microplate reader.

### 4.5. Far-Western Blotting

For far-Western blotting, whole virus lysates and recombinant viral envelope proteins were run on a 12% *v*/*v* SDS-PAGE and electro-transferred to a PVDF membrane. The membrane was blocked overnight with 5% skimmed milk at 4 °C. The following day, the membrane was washed with PBS and then incubated with C1q (20 μg/mL) for 1 h at 4 °C, and 1 h at room temperature. After three washes (10 min each) with PBST, the membrane was probed with rabbit anti-human C1q antibody (1:1000), followed by detection with goat-anti-rabbit IgG HRP (1:1000; Promega, Tokyo, Japan). The blot was developed using SIGMAFAST™ 3,3′-Diaminobenzidine (DAB) (Sigma).

### 4.6. Infection Assay and qRT-PCR Analysis

A549 cells (0.5 × 10^6^) were seeded overnight in growth media. The next day, IAV particles (MOI 1) were pre-incubated with C1q, ghA, ghB, ghC or MBP (20 μg/mL) for 1 h at room temperature and added to A549 cells. After t 2 and 6 h of incubation, the cells were washed with PBS gently and pelleted. cDNA was generated using TaqMan™ High Capacity RNA to cDNA Kit (Applied Biosystems™) from RNA extracted from the cells using GenElute™ Mammalian Total RNA Miniprep Kit (Sigma) along with DNase I (Sigma). qRT-PCR was performed using a StepOnePlus System (Applied Biosystems™, Bedford, MA, USA) at 95 °C for 5 min, followed by 45 cycles of 95 °C for 10 s, 60 °C for 10 s, and 72 °C for 10 s. The relative expression (RQ) was calculated using A549 cells infected with respective untreated viruses as the calibrator. The RQ value was calculated using the formula: RQ = 2^−ΔΔCt^. Primers for each target gene were generated for specificity using the Primer-BLAST software (Basic Local Alignment Search Tool) (http://blast.ncbi.nlm.nih.gov/Blast.cgi; accessed on 29 June 2017) qRT-PCR was performed using primers listed in Table 2, and human 18S rRNA was used as an endogenous control to normalise gene expression.

### 4.7. NF-κB Activation Assay

A549 cells were transfected with a NF-κB reporter plasmid, pNF–κB–Luc (A549–NF-κB–Luc cells) using BioT transfection kit (Bioland Scientific, Paramount, CA, USA). A549–NF-κB–Luc cells were grown to 70% confluence in a 96-well plate. Viral particles (MOI 1) were incubated with 20 µg/mL of C1q, ghA, ghB, ghC, or MBP for 1 h at room temperature and then for another h at 4 °C. The pre-treated viral particles were used to challenge the A549–NF-κB–Luc cells in infection media for 6 h. Virus particles that were not treated with C1q, or treated with MBP alone were used as control. A549–NF-κB–Luc cells treated with recombinant TNF-α (10 ng/ml) and IL-1β (10 ng/ml) (R&D Systems) were used as positive controls for NF-κB activation. Luminescence proportional to the NF-κB activity was measured in relative units using a Clariostar Plus Microplate Reader (BMG Labtech, Ortenberg, Germany).

### 4.8. Production of Pseudo-Typed Lentiviral Particles

HEK 293T cells were grown to 70% confluence and co-transfected using 20 µg of plasmids described in Table 3. Pseudo-typed lentiviral particles expressing HA and NA proteins of IAV were harvested after 48 h by centrifugation at 1500× *g* for 5 min. The cell pellet was treated with lysis buffer (50 mM Tris–HCl pH 7.5, 200 mM NaCl, 5 mM EDTA, 0.1% *v*/*v* Triton X-100). The supernatant and the cell lysate were filtered through a 22 µm filter and centrifuged again at 5000× *g* for 10 min at 4 °C in a closed container. Pseudo-typed lentiviral particles were then concentrated by ultracentrifugation at 25,000× *g* for 3 h at 4 °C [99], and further characterised by Western blotting [47].

### 4.9. Entry Inhibition Assay

First, 50 µL Matched H1N1 or unmatched H3N2 Pseudo-typed lentiviral particles were incubated with 20 µg/mL of C1q, ghA, ghB, ghC or MBP for 1 h at room temperature and for another 1 h at 4 °C. The pre-treated pseudo-typed lentiviral particles were allowed to transduce previously prepared serum-starved MDCK cells (0.1 × 10^5^ cells/well) that reached 50–60% confluence in a 96-well plate for 24 h in serum-free DMEM (Gibco). Next, the medium was replaced with DMEM containing 10% FBS and 1% PS. The plate was incubated for another 48 h for luciferase expression. Luminescence proportional to viral entry was measured in relative units using a Clariostar Plus Microplate Reader (BMG Labtech).

### 4.10. Cell Binding Assay

The ability of IAV virions to bind to A549 cell surface receptors after treatment with C1q was evaluated by performing a cell-binding assay. A549 cells (1 × 10^5^ cells/well) were allowed to adsorb H1N1 or H3N2 (MOI 1), which were pre-treated with or without C1q, recombinant globular heads or MBP (20 µg/mL) at 37 °C for 2 h. Following PBS washes to remove any unbound virions, the cells were fixed with 1% *v*/*v* paraformaldehyde (Fisher Scientific) for 1 min at room temperature. The cells were then subjected to blocking with 2% *w*/*v* BSA for 2 h at 37 °C. The binding was probed with either polyclonal anti-influenza virus H3 (BEI-Resources) or monoclonal anti-influenza H1 (BEI-Resources). The colour was developed by adding 3,3′,5,5′-Tetramethylbenzidine (TMB) substrate, and the reaction was stopped by using 1M H_2_SO_4_. The absorbance was read at 450 nm using a microplate reader.

### 4.11. Statistical Analysis

The graphs were generated using the GraphPad Prism version 9.0.0 for Windows, GraphPad Software, San Diego, CA, USA. The statistical significance was considered as indicated in the figure legends between treated and untreated conditions. Error bars show SD or SEM as stated in the figure legends.

## 5. Conclusions

Based on the M1 mRNA expression analysis, inflammatory cytokine panel, and luciferase reporter-based entry inhibition assay, we propose a model in which C1q, through its globular heads of A, B, and C chains, modulates the efficacy of IAV replication in a subtype-dependent manner by regulating their entry into cells. Since complement mediated neutralisation of IAV only occurs efficiently after the generation of anti-HA antibodies (i.e., after 4 days p.i.), this study provides insight into an alternative mechanism involving C1q that might help protect the host against IAV infections at early stages when the antibodies may yet not be available. Thus, C1q can be developed into an HA and NA-based inhibitor against future H1N1 epidemics and pandemics. However, this would require more research into the specific molecular interactions of C1q against HA and NA and investigations in humanised murine models to better understand the impact of C1q-IAV interaction in the microenvironment of the respiratory system.

These results are in accordance with the previously reported data using the other complement regulators. The regulators of the classical and alternative pathways, Properdin, C4BP and Factor H respectively, were also found to modulate IAV infection in a subtype specific manner, in a manner similar to C1q (47, 77, 100). After treatment of H1N1 particles with factor H, VCP, C4BP, or properdin, A549 cells showed a decrease in M1 expression. However, A549 cells challenged with H3N2 virions that were treated similarly, showed an increase in M1 expression. Hence, the treatment with these proteins resulted in restriction of IAV replication for the H1N1 subtype, while promoting replication for H3N2. The ability of Factor H, VCP, C4BP, or properdin to influence IAV entry in a subtype dependent manner was also established using lentiviral vectors pseudo-typed for H1 + N1 and H3 + N2. The subtype dependent entry modulation was found to occur via interaction with HA, NA, and M1, as seen in the case of C1q. Furthermore, the treatments were found to cause an anti-inflammatory response in the H1N1 subtype while causing a pro-inflammatory response in the H3N2 subtype (1–3). The findings of this study highlight a possible common mechanism that is used by the complement regulatory proteins to modulate the IAV infection in a complement-independent manner. A recent in silico binding analysis between properdin and HA revealed that the binding sites of the top docked poses of properdin with HA of H1N1 and H3N2 were proximal [100]. However, properdin was found to interact with the HA cleavage site of H1N1, but not H3N2 (3). This variation could be attributed to the fact that the trimeric HA structure of H3N2 inhibited the access of properdin to the HA cleavage site (3). A similar interaction pattern between C1q with the HA cleavage site of H1N1 may explain the subtype-dependent entry inhibitor activity observed between H1N1 and H3N2.

## Figures and Tables

**Table 1:** Cytokine response in A549 cells challenged with C1q-, ghA-, ghB-, or ghC-treated H1N1 (**A**) or H3N2 (**B**) virus (“↑” = upregulation; “↓” = downregulation; “−” = no change).

**A**	**C1q**		**ghA**		**ghB**		**ghC**	
	**2 h**	**6 h**	**2 h**	**6 h**	**2 h**	**6 h**	**2 h**	**6 h**
TNF-α	−	↑ (0.15 log_10_)	↑ (2.4 log_10_)	↑ (0.63 log_10_)	↑ (3.20 log_10_)	↑ (0.27 log_10_)	↑ (2.82 log_10_)	↑ (0.03 log_10_)
NFkB	−	↑ (0.09 log_10_)	↑ (0.018 log_10_)	↑ (0.14 log_10_)	↑ (0.33 log_10_)	↑ (0.15 log_10_)	↑ (0.47 log_10_)	↑ (0.04 log_10_)
IFN-α	−	↓ (−0.52 log_10_)	−	↑ (0.06 log_10_)	↓ (0.45 log_10_)	↓ (0.10 log_10_)	↓ (−0.25 log_10_)	↓ (−0.55 log_10_)
IL-6	−	↓ (−0.29 log_10_)	−	↑ (0.50 log_10_)	↑ (1.98 log_10_)	↑ (0.18 log_10_)	↑ (1.84 log_10_)	↓ (−0.05 log_10_)
IL-12	−	↑ (0.51 log_10_)	↓ (−0.93 log_10_)	↑ (1.17 log_10_)	↓ (−0.14 log_10_)	↑ (0.34 log_10_)	↑ (0.19 log_10_)	↑ (0.23 log_10_)
RANTES	−	↓ (−0.018 log_10_)	↓ (−0.39 log_10_)	↑ (0.17 log_10_)	↓ (−0.40 log_10_)	↑ (0.23 log_10_)	↑ (0.04 log_10_)	↓ (−0.52 log_10_)
**B**	**C1q**		**ghA**		**ghB**		**ghC**	
	**2 h**	**6 h**	**2 h**	**6 h**	**2 h**	**6 h**	**2 h**	**6 h**
TNF-α	↓ (−0.32 log_10_)	↑ (0.37 log_10_)	↓ (−0.29 log_10_)	↑ (0.04 log_10_)	↑ (0.31 log_10_)	↓ (−0.25 log_10_)	↓ (−0.11 log_10_)	↑ (0.39 log_10_)
NF-κB	↓ (−0.15 log_10_)	↓ (−0.20 log_10_)	↑ (0.77 log_10_)	↓ (−0.42 log_10_)	↑ (0.60 log_10_)	↓ (−0.21 log_10_)	↑ (0.65 log_10_)	↓ (−0.04 log_10_)
IFN-α	↓ (−2.12 log_10_)	↓ (−0.07 log_10_)	↓ (−0.96 log_10_)	↓ (−1.88 log_10_)	↓ (−2.15 log_10_)	↓ (−0.23 log_10_)	↓ (−0.51 log_10_)	↓ (−1.75 log_10_)
IL-6	↑ (0.06 log_10_)	↓ (−0.32 log_10_)	↑ (0.30 log_10_)	↓ (−0.83 log_10_)	↑ (0.29 log_10_)	↓ (−0.69 log_10_)	↓ (−0.08 log_10_)	↑ (0.25 log_10_)
IL-12	−	↑ (1.5 log_10_)	−	↓ (−0.39 log_10_)	−	↑ (0.71 log_10_)	−	↓ (−0.24 log_10_)
RANTES	↓ (−3.20 log_10_)	↓ (−0.01 log_10_)	↑ (−1.4 log_10_)	↓ (−0.74 log_10_)	↓ (−0.89 log_10_)	↓ (−0.29 log_10_)	↑ (0.35 log_10_)	↓ (−1.36 log_10_)

**Table 2:** Forward and reverse primers used for qRT-PCR.

Target	Forward Primer	Reverse Primer
18S	5′-ATGGCCGTTC TTAGTTGGTG-3′	5′-CGCTGAGCCA GTCAGTGTAG-3′
IL-6	5′-GAAAGCAGCA AAGAGGCACT-3′	5′-TTTCACCAGG CAAGTCTCCT-3′
IL-12	5′-AACTTGCAGC TGAAGCCATT-3′	5′-GACCTGAACG CAGAATGTCA-3′
TNF-α	5′-AGCCCATGTT GTAGCAAACC-3′	5′-TGAGGTACAG GCCCTCTGAT-3′
M1	5′AAACATATGTCTGATAAC GAAGGAGAACAGTTCTT-3′	5′GCTGAATTCTACCT CATGGTCTTCTTGA-3′
RANTES	5′-GCGGGTACCAT GAAGATCTCTG-3′	5′-GGGTCAGAATC AAGAAACCCTC-3′
IFN-α	5′-TTT CTC CTG CC T GAA GGA CAG-3′	5′-GCT CAT GAT TTC TGC TCT GAC A-3′

**Table 3:** Plasmids used for pseudo-typed lentivirus particle production [77].

	H1N1	H3N2	VSV-G
Envelope Protein-Coding Plasmid	pcDNA3.1-swineH1-flag (H1 from swine H1N1 A/California/04/09) (Codon optimized H1 (Genecust))	pcDNA-H3 (H3 from A/Denmark/70/03/(H3N2)) (Codon optimized H3 (Geneart))	pCMV-VSV-G (Addgene plasmid # 8454)
	pcDNA3.1-swine N1-flag (N1 from swine H1N1 A/California/04/09) (Codon optimised N1 (Genecust))	pI.18-N2 (N2 from human H3N2 A/Texas/50/2012)	
Backbone Plasmid	pHIV–Luciferase (Addgene plasmid # 21375)		
Packaging Plasmid	psPAX2 (Addgene plasmid # 12260)		

## Supplementary Materials

The following supporting information can be downloaded at: https://www.mdpi.com/article/10.3390/ijms23063045/s1. Supporting information consisting of a blot testing cross reactivity of anti-C1q against IAV lysate, C1q and recombinant globular head cytotoxicity panel and the cytokine panel.

## References

[1] Simonsen L., Viboud C., Taylor R.J., Miller M.A., Rappuoli R., Del Giudice G. (2011). The Epidemiology of Influenza and Its Control. Influenza Vaccines for the Future.

[2] Bresee J.S., Fry A.M., Sambhara S., Cox N.J., Plotkin S.A., Orenstein W.A., Offit P.A., Edwards K.M. (2018). Inactivated Influenza Vaccines. Plotkin’s Vaccines.

[3] Taubenberger J.K., Morens D.M. (2008). The Pathology of Influenza Virus Infections. Annu. Rev. Pathol. Mech. Dis..

[4] García-Sastre A., Schaechter M. (2009). Influenza. Encyclopedia of Microbiology.

[5] Tong S., Zhu X., Li Y., Shi M., Zhang J., Bourgeois M., Yang H., Chen X., Recuenco S., Gomez J. (2013). New world bats harbor diverse influenza A viruses. PLoS Pathog..

[6] CDC The 2009 H1N1 Pandemic: Summary Highlights, April 2009–April 2010. https://www.cdc.gov/h1n1flu/cdcresponse.htm.

[7] Samji T. (2009). Influenza A: Understanding the viral life cycle. Yale J. Biol. Med..

[8] Leung H.S., Li O.T., Chan R.W., Chan M.C., Nicholls J.M., Poon L.L. (2012). Entry of influenza A Virus with a alpha2,6-linked sialic acid binding preference requires host fibronectin. J. Virol..

[9] Steinhauer D., Wharton S., Nicholson K.G., Webster R.G., Hay A.J. (1998). Structure and Function of the Haemagglutinin. Textbook of Influenza.

[10] Wilson I.A., Skehel J.J., Wiley D.C. (1981). Structure of the haemagglutinin membrane glycoprotein of influenza virus at 3 Å resolution. Nature.

[11] Skehel J.J., Wiley D.C. (2000). Receptor Binding and Membrane Fusion in Virus Entry: The Influenza Hemagglutinin. Annu. Rev. Biochem..

[12] Wiley D.C., Wilson I.A., Skehel J.J. (1981). Structural identification of the antibody-binding sites of Hong Kong influenza haemagglutinin and their involvement in antigenic variation. Nature.

[13] Shtyrya Y.A., Mochalova L.V., Bovin N.V. (2009). Influenza virus neuraminidase: Structure and function. Acta Nat..

[14] Matrosovich M.N., Matrosovich T.Y., Gray T., Roberts N.A., Klenk H.-D. (2004). Neuraminidase Is Important for the Initiation of Influenza Virus Infection in Human Airway Epithelium. J. Virol..

[15] Holsinger L.J., Alams R. (1991). Influenza virus M2 integral membrane protein is a homotetramer stabilized by formation of disulfide bonds. Virology.

[16] Martin K., Helenius A. (1991). Transport of incoming influenza virus nucleocapsids into the nucleus. J. Virol..

[17] Sieczkarski S.B., Whittaker G.R., Marsh M. (2005). Viral Entry. Membrane Trafficking in Viral Replication.

[18] Stegmann T. (2000). Membrane Fusion Mechanisms: The Influenza Hemagglutinin Paradigm and its Implications for Intracellular Fusion. Traffic.

[19] Cao X. (2016). Self-regulation and cross-regulation of pattern-recognition receptor signalling in health and disease. Nat. Rev. Immunol..

[20] Ouyang J., Zhu X., Chen Y., Wei H., Chen Q., Chi X., Qi B., Zhang L., Zhao Y., Gao G.F. (2014). NRAV, a Long Noncoding RNA, Modulates Antiviral Responses through Suppression of Interferon-Stimulated Gene Transcription. Cell Host Microbe.

[21] Chen X., Liu S., Goraya M.U., Maarouf M., Huang S., Chen J.-L. (2018). Host Immune Response to Influenza A Virus Infection. Front. Immunol..

[22] Kumar H., Kawai T., Akira S. (2011). Pathogen Recognition by the Innate Immune System. Int. Rev. Immunol..

[23] Kawai T., Akira S. (2010). The role of pattern-recognition receptors in innate immunity: Update on Toll-like receptors. Nat. Immunol..

[24] Takeuchi O., Akira S. (2010). Pattern Recognition Receptors and Inflammation. Cell.

[25] Philpott D.J., Sorbara M.T., Robertson S.J., Croitoru K., Girardin S.E. (2014). NOD proteins: Regulators of inflammation in health and disease. Nat. Rev. Immunol..

[26] Munir M. (2010). TRIM Proteins: Another Class of Viral Victims. Sci. Signal..

[27] Yoneyama M., Onomoto K., Jogi M., Akaboshi T., Fujita T. (2015). Viral RNA detection by RIG-I-like receptors. Curr. Opin. Immunol..

[28] Hiscott J., Lin R., Nakhaei P., Paz S. (2006). MasterCARD: A priceless link to innate immunity. Trends Mol. Med..

[29] Schulz O., Diebold S.S., Chen M., Näslund T.I., Nolte M.A., Alexopoulou L., Azuma Y.-T., Flavell R.A., Liljeström P., Reis e Sousa C. (2005). Toll-like receptor 3 promotes cross-priming to virus-infected cells. Nature.

[30] Goubau D., Schlee M., Deddouche S., Pruijssers A.J., Zillinger T., Goldeck M., Schuberth C., Van der Veen A.G., Fujimura T., Rehwinkel J. (2014). Antiviral immunity via RIG-I-mediated recognition of RNA bearing 5′-diphosphates. Nature.

[31] Ablasser A., Poeck H., Anz D., Berger M., Schlee M., Kim S., Bourquin C., Goutagny N., Jiang Z., Fitzgerald K.A. (2009). Selection of Molecular Structure and Delivery of RNA Oligonucleotides to Activate TLR7 versus TLR8 and to Induce High Amounts of IL-12p70 in Primary Human Monocytes. J. Immunol..

[32] Lund J.M., Alexopoulou L., Sato A., Karow M., Adams N.C., Gale N.W., Iwasaki A., Flavell R.A. (2004). Recognition of single-stranded RNA viruses by Toll-like receptor 7. Proc. Natl. Acad. Sci. USA.

[33] Pothlichet J., Meunier I., Davis B.K., Ting J.P.Y., Skamene E., von Messling V., Vidal S.M. (2013). Type I IFN Triggers RIG-I/TLR3/NLRP3-dependent Inflammasome Activation in Influenza A Virus Infected Cells. PLoS Pathog..

[34] Guarda G., Zenger M., Yazdi A.S., Schroder K., Ferrero I., Menu P., Tardivel A., Mattmann C., Tschopp J. (2011). Differential Expression of NLRP3 among Hematopoietic Cells. J. Immunol..

[35] Martinon F., Mayor A., Tschopp J. (2009). The Inflammasomes: Guardians of the Body. Annu. Rev. Immunol..

[36] Ichinohe T., Pang I.K., Iwasaki A. (2010). Influenza virus activates inflammasomes via its intracellular M2 ion channel. Nat. Immunol..

[37] McAuley J.L., Tate M.D., MacKenzie-Kludas C.J., Pinar A., Zeng W., Stutz A., Latz E., Brown L.E., Mansell A. (2013). Activation of the NLRP3 Inflammasome by IAV Virulence Protein PB1-F2 Contributes to Severe Pathophysiology and Disease. PLoS Pathog..

[38] Guo H., Kumar P., Malarkannan S. (2011). Evasion of natural killer cells by influenza virus. J. Leukoc. Biol..

[39] van Helden M.J.G., de Graaf N., Boog C.J.P., Topham D.J., Zaiss D.M.W., Sijts A.J.A.M. (2012). The Bone Marrow Functions as the Central Site of Proliferation for Long-Lived NK Cells. J. Immunol..

[40] Mendelson M., Tekoah Y., Zilka A., Gershoni-Yahalom O., Gazit R., Achdout H., Bovin N.V., Meningher T., Mandelboim M., Mandelboim O. (2010). NKp46 O-Glycan Sequences That Are Involved in the Interaction with Hemagglutinin Type 1 of Influenza Virus. J. Virol..

[41] Heer A.K., Harris N.L., Kopf M., Marsland B.J. (2008). CD4^+^and CD8^+^ T Cells Exhibit Differential Requirements for CCR7-Mediated Antigen Transport during Influenza Infection. J. Immunol..

[42] Hintzen G., Ohl L., del Rio M.-L., Rodriguez-Barbosa J.-I., Pabst O., Kocks J.R., Krege J., Hardtke S., Förster R. (2006). Induction of Tolerance to Innocuous Inhaled Antigen Relies on a CCR7-Dependent Dendritic Cell-Mediated Antigen Transport to the Bronchial Lymph Node. J. Immunol..

[43] GeurtsvanKessel C.H., Willart M.A.M., van Rijt L.S., Muskens F., Kool M., Baas C., Thielemans K., Bennett C., Clausen B.E., Hoogsteden H.C. (2008). Clearance of influenza virus from the lung depends on migratory langerin ^+^CD11b^−^ but not plasmacytoid dendritic cells. J. Exp. Med..

[44] Van de Sandt C.E., Kreijtz J.H.C.M., Rimmelzwaan G.F. (2012). Evasion of Influenza A Viruses from Innate and Adaptive Immune Responses. Viruses.

[45] GeurtsvanKessel C.H., Bergen I.M., Muskens F., Boon L., Hoogsteden H.C., Osterhaus A.D.M.E., Rimmelzwaan G.F., Lambrecht B.N. (2009). Both Conventional and Interferon Killer Dendritic Cells Have Antigen-Presenting Capacity during Influenza Virus Infection. PLoS ONE.

[46] Tumpey T.M., García-Sastre A., Taubenberger J.K., Palese P., Swayne D.E., Pantin-Jackwood M.J., Schultz-Cherry S., Solórzano A., Rooijen N.V., Katz J.M. (2005). Pathogenicity of Influenza Viruses with Genes from the 1918 Pandemic Virus: Functional Roles of Alveolar Macrophages and Neutrophils in Limiting Virus Replication and Mortality in Mice. J. Virol..

[47] Murugaiah V., Varghese P.M., Saleh S.M., Tsolaki A.G., Alrokayan S.H., Khan H.A., Collison K.S., Sim R.B., Nal B., Al-Mohanna F.A. (2020). Complement-Independent Modulation of Influenza A Virus Infection by Factor H. Front. Immunol..

[48] Sim R.B., Tsiftsoglou S.A. (2004). Proteases of the complement system. Biochem. Soc. Trans..

[49] Woodruff T.M., Nandakumar K.S., Tedesco F. (2011). Inhibiting the C5–C5a receptor axis. Mol. Immunol..

[50] Muller-Eberhard H.J. (1986). The Membrane Attack Complex of Complement. Annu. Rev. Immunol..

[51] Kopf M., Abel B., Gallimore A., Carroll M., Bachmann M.F. (2002). Complement component C3 promotes T-cell priming and lung migration to control acute influenza virus infection. Nat. Med..

[52] Hicks J.T., Ennis F.A., Kim E., Verbonitz M. (1978). The Importance of an Intact Complement Pathway in Recovery from a Primary Viral Infection: Influenza in Decomplemented and in C5-Deficient Mice. J. Immunol..

[53] Kandasamy M., Ying P.C., Ho A.W.S., Sumatoh H.R., Schlitzer A., Hughes T.R., Kemeny D.M., Morgan B.P., Ginhoux F., Sivasankar B. (2013). Complement Mediated Signaling on Pulmonary CD103+ Dendritic Cells Is Critical for Their Migratory Function in Response to Influenza Infection. PLoS Pathog..

[54] Carroll M.C., Isenman D.E. (2012). Regulation of Humoral Immunity by Complement. Immunity.

[55] Jayasekera J.P., Moseman E.A., Carroll M.C. (2007). Natural Antibody and Complement Mediate Neutralization of Influenza Virus in the Absence of Prior Immunity. J. Virol..

[56] Rattan A., Pawar S.D., Nawadkar R., Kulkarni N., Lal G., Mullick J., Sahu A. (2017). Synergy between the classical and alternative pathways of complement is essential for conferring effective protection against the pandemic influenza A(H1N1) 2009 virus infection. PLoS Pathog..

[57] Mozdzanowska K., Feng J., Eid M., Zharikova D., Gerhard W. (2006). Enhancement of neutralizing activity of influenza virus-specific antibodies by serum components. Virology.

[58] Feng J.Q., Mozdzanowska K., Gerhard W. (2002). Complement component C1q enhances the biological activity of influenza virus hemagglutinin-specific antibodies depending on their fine antigen specificity and heavy-chain isotype. J. Virol..

[59] Zhang J., Li G., Liu X., Wang Z., Liu W., Ye X. (2009). Influenza A virus M1 blocks the classical complement pathway through interacting with C1qA. J. Gen. Virol..

[60] Castellano G., Woltman A.M., Nauta A.J., Roos A., Trouw L.A., Seelen M.A., Schena F.P., Daha M.R., van Kooten C. (2004). Maturation of dendritic cells abrogates C1q production In Vivo and In Vitro. Blood.

[61] Day N.K., Gewurz H., Pickering R.J., Good R.A. (1970). Ontogenetic Development of Cl_q_ Synthesis in the Piglet. J. Immunol..

[62] Kaul M., Loos M. (2001). Expression of membrane C1q in human monocyte-derived macrophages is developmentally regulated and enhanced by interferon-γ. FEBS Lett..

[63] Petry F., Botto M., Holtappels R., Walport M.J., Loos M. (2001). Reconstitution of the Complement Function in C1q-Deficient (C1qa^−/−^) Mice with Wild-Type Bone Marrow Cells. J. Immunol..

[64] Stecher V.J., Morse J.H., Thorbecke G.J. (1967). Sites of Production of Primate Serum Proteins Associated with the Complement System. Proc. Soc. Exp. Biol. Med..

[65] Kishore U., Gaboriaud C., Waters P., Shrive A.K., Greenhough T.J., Reid K.B.M., Sim R.B., Arlaud G.J. (2004). C1q and tumor necrosis factor superfamily: Modularity and versatility. Trends Immunol..

[66] Kishore U., Reid K.B.M. (2000). C1q: Structure, function, and receptors. Immunopharmacology.

[67] Thielens N.M., Tacnet-Delorme P., Arlaud G.J. (2002). Interaction of C1q and Mannan-Binding Lectin with Viruses. Immunobiology.

[68] Fausther-Bovendo H., Vieillard V., Sagan S., Bismuth G., Debré P. (2010). HIV gp41 Engages gC1qR on CD4^+^ T Cells to Induce the Expression of an NK Ligand through the PIP3/H_2_O_2_ Pathway. PLoS Pathog..

[69] Ebenbichler C.F., Thielens N.M., Vornhagen R., Marschang P., Arlaud G.J., Dierich M.P. (1991). Human immunodeficiency virus type 1 activates the classical pathway of complement by direct C1 binding through specific sites in the transmembrane glycoprotein gp41. J. Exp. Med..

[70] Thielens N.M., Bally I.M., Ebenbichler C.F., Dierich M.P., Arlaud G.J. (1993). Further characterization of the interaction between the C1q subcomponent of human C1 and the transmembrane envelope glycoprotein gp41 of HIV-1. J. Immunol..

[71] Kishore U., Gupta S.K., Perdikoulis M.V., Kojouharova M.S., Urban B.C., Reid K.B.M. (2003). Modular Organization of the Carboxyl-Terminal, Globular Head Region of Human C1q A, B, and C Chains. J. Immunol..

[72] Kittlesen D.J., Chianese-Bullock K.A., Yao Z.Q., Braciale T.J., Hahn Y.S. (2000). Interaction between complement receptor gC1qR and hepatitis C virus core protein inhibits T-lymphocyte proliferation. J. Clin. Investig..

[73] Mohan K.V.K., Ghebrehiwet B., Atreya C.D. (2002). The N-terminal conserved domain of rubella virus capsid interacts with the C-terminal region of cellular p32 and overexpression of p32 enhances the viral infectivity. Virus Res..

[74] Matthews D.A., Russell W.C. (1998). Adenovirus core protein V interacts with p32--a protein which is associated with both the mitochondria and the nucleus. J. Gen. Virol..

[75] Wang Y., Finan J.E., Middeldorp J.M., Hayward S.D. (1997). P32/TAP, a Cellular Protein That Interacts with EBNA-1 of Epstein–Barr Virus. Virology.

[76] Kunnakkadan U., Nag J., Kumar N.A., Mukesh R.K., Suma S.M., Johnson J.B., Dutch R.E. (2019). Complement-Mediated Neutralization of a Potent Neurotropic Human Pathogen, Chandipura Virus, Is Dependent on C1q. J. Virol..

[77] Varghese P.M., Murugaiah V., Beirag N., Temperton N., Khan H.A., Alrokayan S.H., Al-Ahdal M.N., Nal B., Al-Mohanna F.A., Sim R.B. (2021). C4b Binding Protein Acts as an Innate Immune Effector Against Influenza A Virus. Front. Immunol..

[78] La Gruta N.L., Kedzierska K., Stambas J., Doherty P.C. (2007). A question of self-preservation: Immunopathology in influenza virus infection. Immunol. Cell Biol..

[79] Shinya K., Gao Y., Cilloniz C., Suzuki Y., Fujie M., Deng G., Zhu Q., Fan S., Makino A., Muramoto Y. (2012). Integrated Clinical, Pathologic, Virologic, and Transcriptomic Analysis of H5N1 Influenza Virus-Induced Viral Pneumonia in the Rhesus Macaque. J. Virol..

[80] Ludwig S., Planz O. (2008). Influenza viruses and the NF-kappaB signaling pathway—Towards a novel concept of antiviral therapy. Biol. Chem..

[81] Oslund K.L., Baumgarth N. (2011). Influenza-induced innate immunity: Regulators of viral replication, respiratory tract pathology & adaptive immunity. Future Virol..

[82] Lee S.M., Cheung C.Y., Nicholls J.M., Hui K.P., Leung C.Y., Uiprasertkul M., Tipoe G.L., Lau Y.L., Poon L.L., Ip N.Y. (2008). Hyperinduction of cyclooxygenase-2-mediated proinflammatory cascade: A mechanism for the pathogenesis of avian influenza H5N1 infection. J. Infect. Dis..

[83] Yarilina A., Park-Min K.H., Antoniv T., Hu X., Ivashkiv L.B. (2008). TNF activates an IRF1-dependent autocrine loop leading to sustained expression of chemokines and STAT1-dependent type I interferon-response genes. Nat. Immunol..

[84] Schneider W.M., Chevillotte M.D., Rice C.M. (2014). Interferon-Stimulated Genes: A Complex Web of Host Defenses. Annu. Rev. Immunol..

[85] Al-Ahdal M.N., Murugaiah V., Varghese P.M., Abozaid S.M., Saba I., Al-Qahtani A.A., Pathan A.A., Kouser L., Nal B., Kishore U. (2018). Entry Inhibition and Modulation of Pro-Inflammatory Immune Response Against Influenza A Virus by a Recombinant Truncated Surfactant Protein D. Front. Immunol..

[86] Kim K.S., Jung H., Shin I.K., Choi B.-R., Kim D.H. (2015). Induction of interleukin-1 beta (IL-1β) is a critical component of lung inflammation during influenza A (H1N1) virus infection. J. Med. Virol..

[87] Ramos I., Bernal-Rubio D., Durham N., Belicha-Villanueva A., Lowen A.C., Steel J., Fernandez-Sesma A. (2011). Effects of Receptor Binding Specificity of Avian Influenza Virus on the Human Innate Immune Response. J. Virol..

[88] Ramos I., Fernandez-Sesma A. (2015). Modulating the Innate Immune Response to Influenza A Virus: Potential Therapeutic Use of Anti-Inflammatory Drugs. Front. Immunol..

[89] Muramoto Y., Shoemaker J.E., Le M.Q., Itoh Y., Tamura D., Sakai-Tagawa Y., Imai H., Uraki R., Takano R., Kawakami E. (2014). Disease Severity Is Associated with Differential Gene Expression at the Early and Late Phases of Infection in Nonhuman Primates Infected with Different H5N1 Highly Pathogenic Avian Influenza Viruses. J. Virol..

[90] Cilloniz C., Pantin-Jackwood M.J., Ni C., Goodman A.G., Peng X., Proll S.C., Carter V.S., Rosenzweig E.R., Szretter K.J., Katz J.M. (2010). Lethal Dissemination of H5N1 Influenza Virus Is Associated with Dysregulation of Inflammation and Lipoxin Signaling in a Mouse Model of Infection. J. Virol..

[91] Peiris J.S.M., Cheung C.Y., Leung C.Y.H., Nicholls J.M. (2009). Innate immune responses to influenza A H5N1: Friend or foe?. Trends Immunol..

[92] Tisoncik J.R., Korth M.J., Simmons C.P., Farrar J., Martin T.R., Katze M.G. (2012). Into the Eye of the Cytokine Storm. Microbiol. Mol. Biol. Rev..

[93] Duvigneau S., Sharma-Chawla N., Boianelli A. (2016). Hierarchical effects of pro-inflammatory cytokines on the post-influenza susceptibility to pneumococcal coinfection. Sci. Rep..

[94] Cheng X.-W., Lu J., Wu C.-L., Yi L.-N., Xie X., Shi X.-D., Fang S.-S., Zan H., Kung H.-f., He M.-L. (2011). Three fatal cases of pandemic 2009 influenza A virus infection in Shenzhen are associated with cytokine storm. Respir. Physiol. Neurobiol..

[95] Dawson T.C., Beck M.A., Kuziel W.A., Henderson F., Maeda N. (2000). Contrasting Effects of CCR5 and CCR2 Deficiency in the Pulmonary Inflammatory Response to Influenza A Virus. Am. J. Pathol..

[96] Kaufmann A., Salentin R., Meyer R.G., Bussfeld D., Pauligk C., Fesq H., Hofmann P., Nain M., Gemsa D., Sprenger H. (2001). Defense against Influenza A Virus Infection: Essential Role of the Chemokine System. Immunobiology.

[97] Tan L.A., Yu B., Sim F.C.J., Kishore U., Sim R.B. (2010). Complement activation by phospholipids: The interplay of factor H and C1q. Protein Cell.

[98] Klimov A., Balish A., Veguilla V., Sun H., Schiffer J., Lu X., Katz J.M., Hancock K., Kawaoka Y., Neumann G. (2012). Influenza Virus Titration, Antigenic Characterization, and Serological Methods for Antibody Detection. Influenza Virus: Methods and Protocols.

[99] Tang D.-J., Lam Y.-M., Siu Y.-L., Lam C.-H., Chu S.-L., Peiris J.S.M., Buchy P., Nal B., Bruzzone R. (2012). A Single Residue Substitution in the Receptor-Binding Domain of H5N1 Hemagglutinin Is Critical for Packaging into Pseudotyped Lentiviral Particles. PLoS ONE.

[100] Varghese P.M., Mukherjee S., Al-Mohanna F.A., Saleh S.M., Almajhdi F.N., Beirag N., Alkahtani S.H., Rajkumari R., Rogier B.N., Sim R.B. (2021). Human Properdin Released by Infiltrating Neutrophils Can Modulate Influenza A Virus Infection. Front. Immunol..

