# Supplementary material for: Human C1q Regulates Influenza A Virus Infection and Inflammatory Response via Its Globular Domain
Source: Int J Mol Sci. 2022 Mar 11;23(6):3045. doi: 10.3390/ijms23063045 (PMC8949502; doi:10.3390/ijms23063045)
Supplement: Supplementary file 1 [file ijms-23-03045-s001.zip › ijms-1538446-supplementary.pdf]

Supplementary Figures

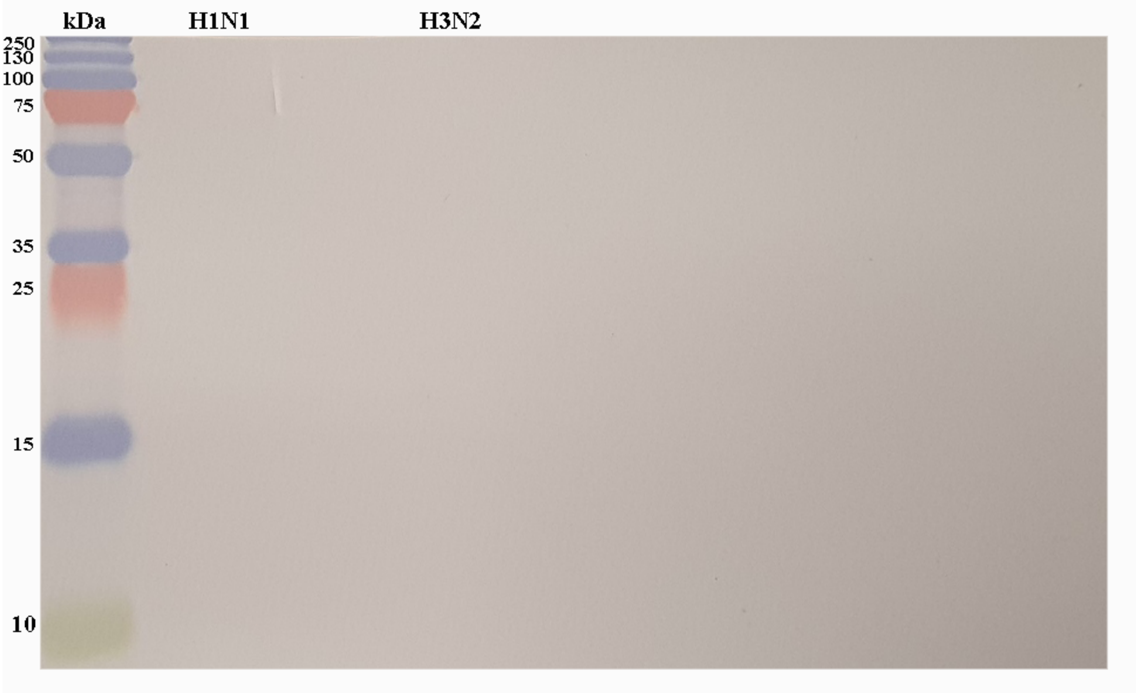

Figure S1: **No non-specific interaction between the anti-human C1q antibody and the IAV viral lysate were observed**

H1N1, H3N2 virus lysates were separated using SDS-PAGE (12% w/v) under reducing conditions and then transferred onto an activated PVDF membrane. Following the blocking process with PBS+5% w/v BSA. After PBS washes, the membrane was probed with rabbit anti-human C1q antibody (1:1000). No cross reactivity was observed between the anti-C1q antibody and the proteins in the IAV lysate.

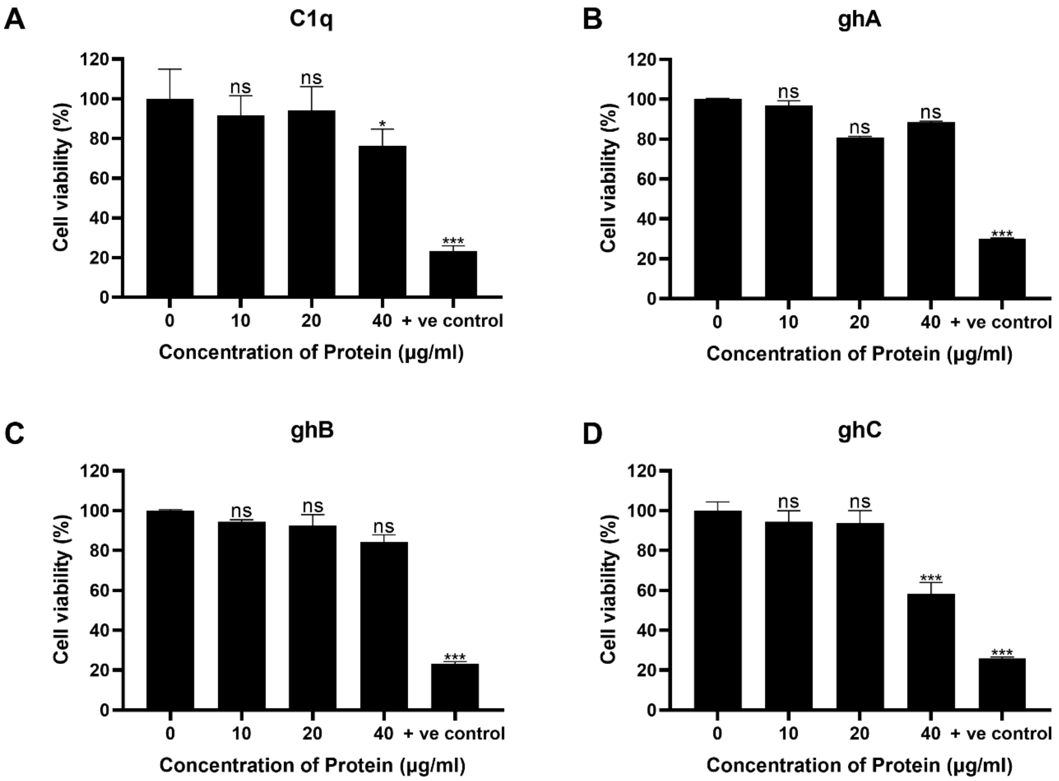

Figure S2: **Treatment with C1q or its recombinant globular heads does not affect A549 cell viability at concentration up to 20µg/ml.**

3-(4,5-dimethylthiazol-2,5-diphenyltetrazolium bromide (MTT) assay was performed by seeding A549 cells (1×10^4 cells/well) in a 96-well microtiter plate. The monolayers formed were treated for 24h with either 10, 20, 40 µg/ml of C1q (A) or its recombinant globular heads ghA (B), ghB (C) or ghC (D), under anaerobic conditions at 37°C. Samples that were treated with a similar volume of protein vehicle were used as control samples. Samples that were treated with a 2mM of Staurosporine were used as positive control samples The assays were performed following the manufacturer's recommendations, and cell viability was measured by determining the absorbance at 570 nm. The background was subtracted from all data points. The data obtained were normalised with 100% cell viability being defined as the mean of the absorbance recorded from the control sample. The data were presented as the mean of the normalised duplicates ±. Significance was determined using the two-way ANOVA (n = 2). No significant difference in cell viability was observed between the samples treated with C1q or its recombinant globular heads at 20 µg/ml. However ~25% and 42% reduction of cell viability was observed in A549 cells treated with 40 µg/ml of C1q and ghC respectively.

H1N1 - C1q

A

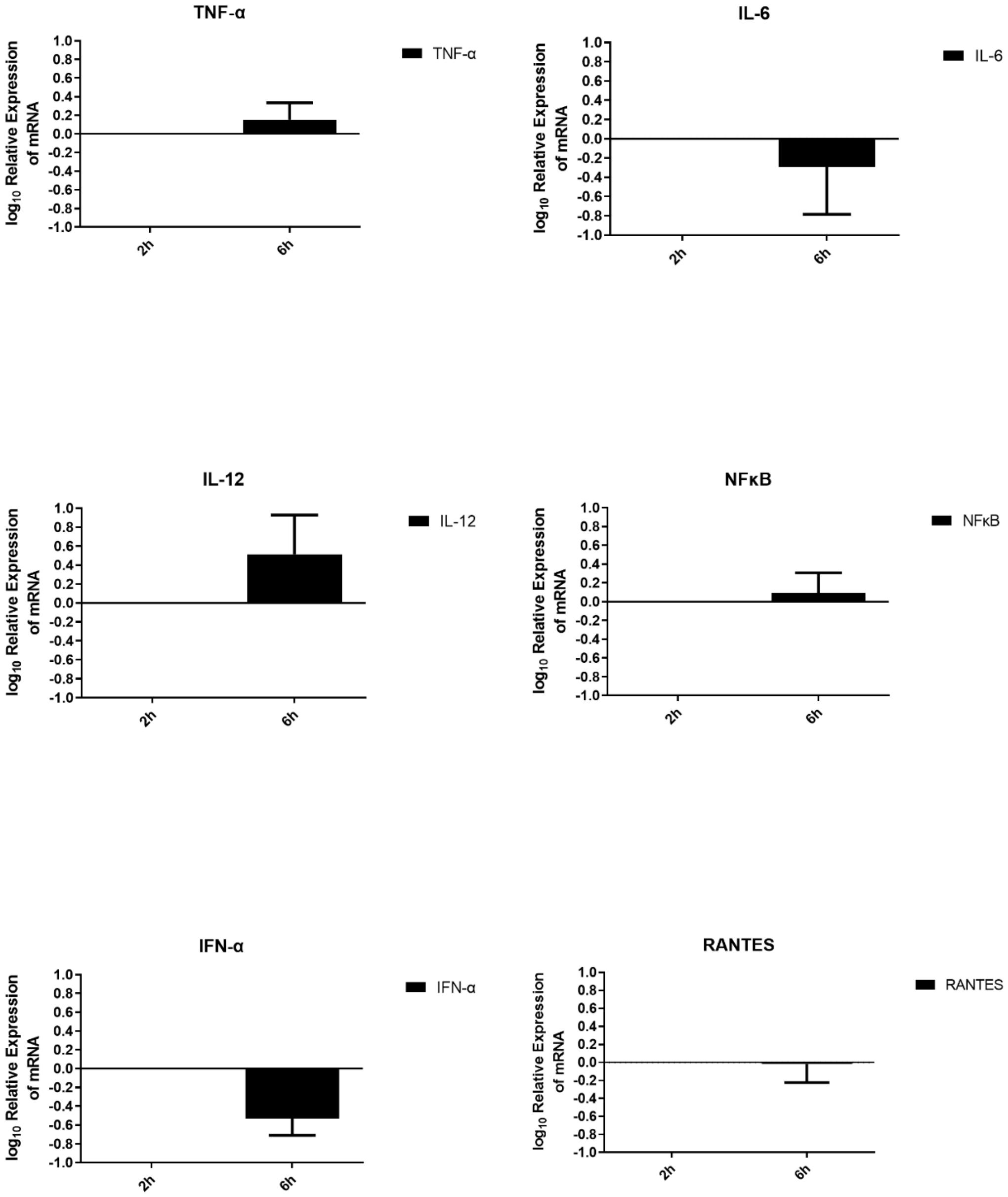

TNF-α
IL-6
IL-12
NFκB
IFN-α
RANTES
log10 Relative Expression of mRNA
1.0
0.8
0.6
0.4
0.2
0.0
-0.2
-0.4
-0.6
-0.8
-1.0
2h
6h

B

H3N2 - C1q

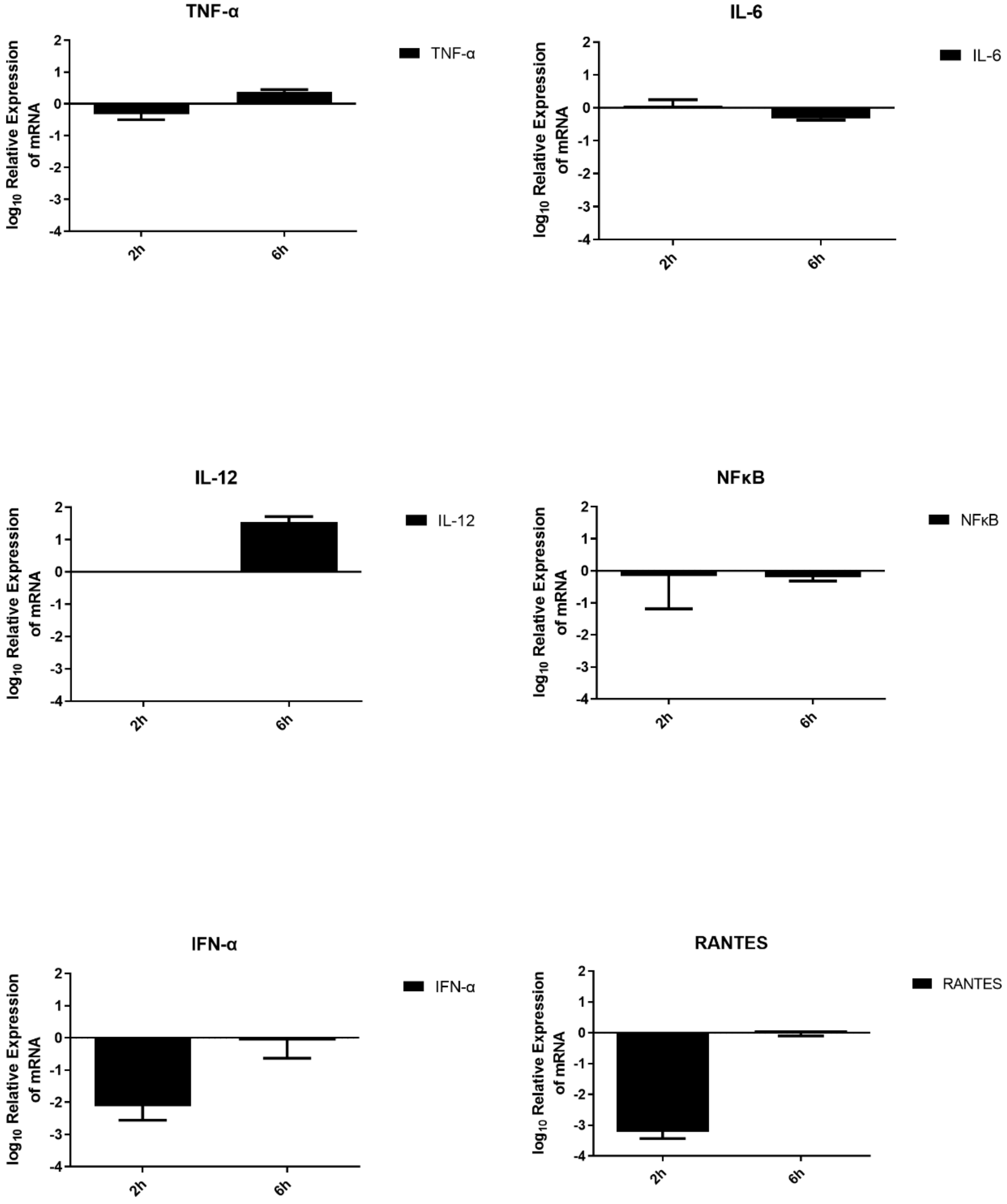

TNF-α
IL-6
IL-12
NFκB
IFN-α
RANTES
$\log_{10}$ Relative Expression of mRNA
2h
6h
2
1
0
-1
-2
-3
-4

H1N1 - ghA

C

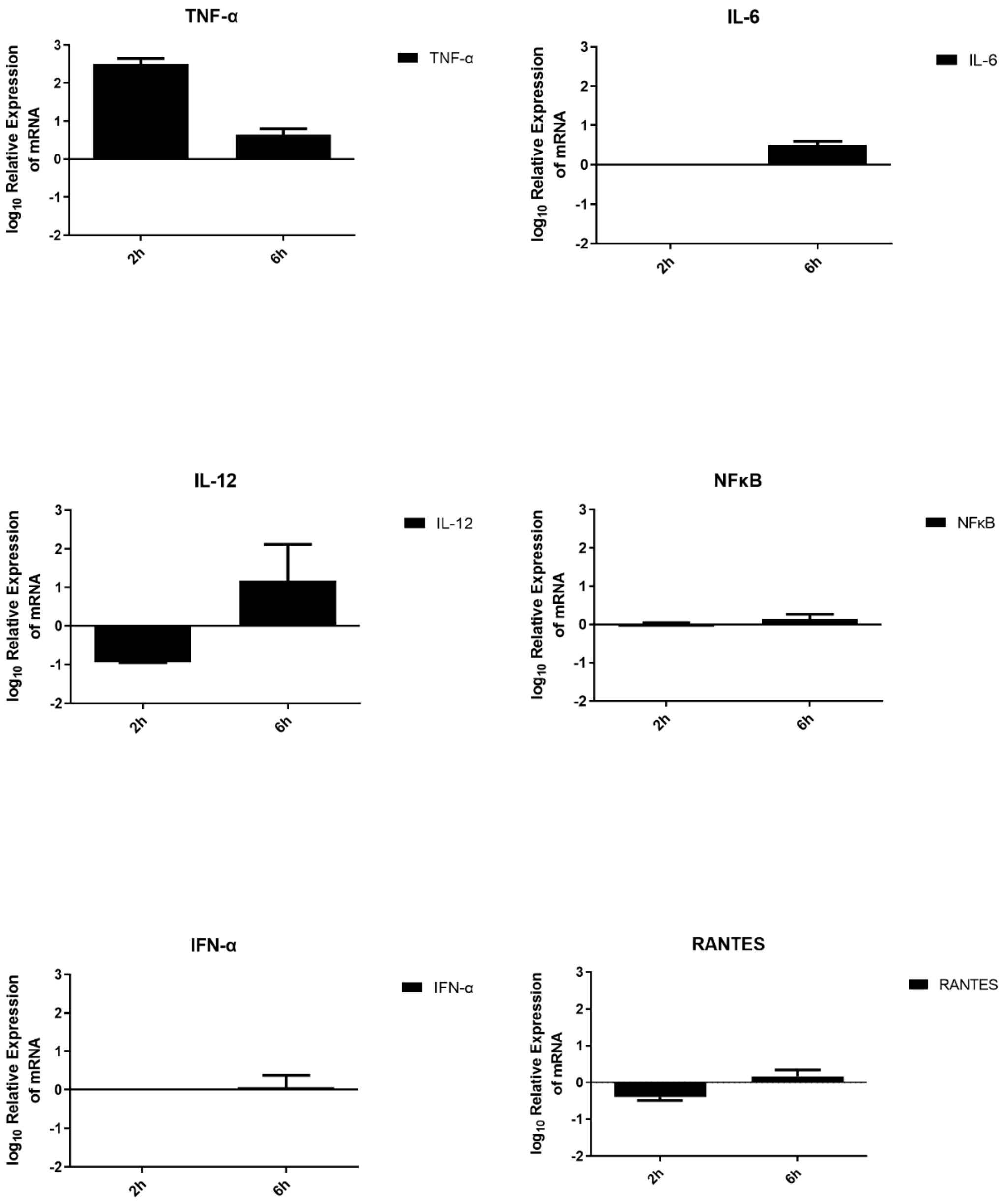
TNF-α
TNF-α
IL-6
IL-6
IL-12
IL-12
NFκB
NFκB
IFN-α
IFN-α
RANTES
RANTES
$\log_{10}$ Relative Expression of mRNA
2h
6h
3
2
1
0
-1
-2

H3N2 - ghA

D

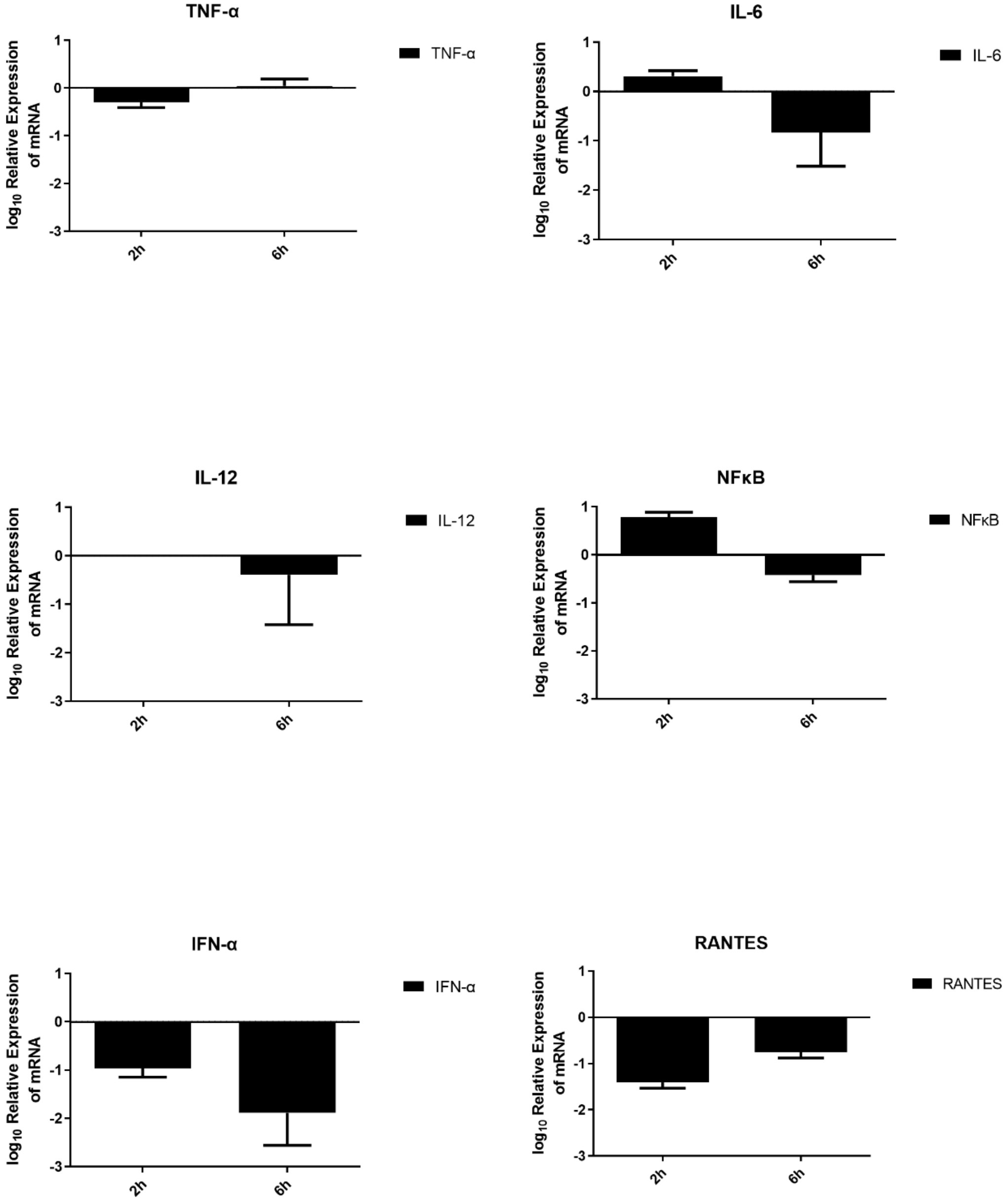

TNF-α
log10 Relative Expression of mRNA
1
0
-1
-2
-3
2h
6h
TNF-α
IL-6
IL-6
IL-12
IL-12
NFκB
NFκB
IFN-α
IFN-α
RANTES
RANTES

H1N1 - ghB

E

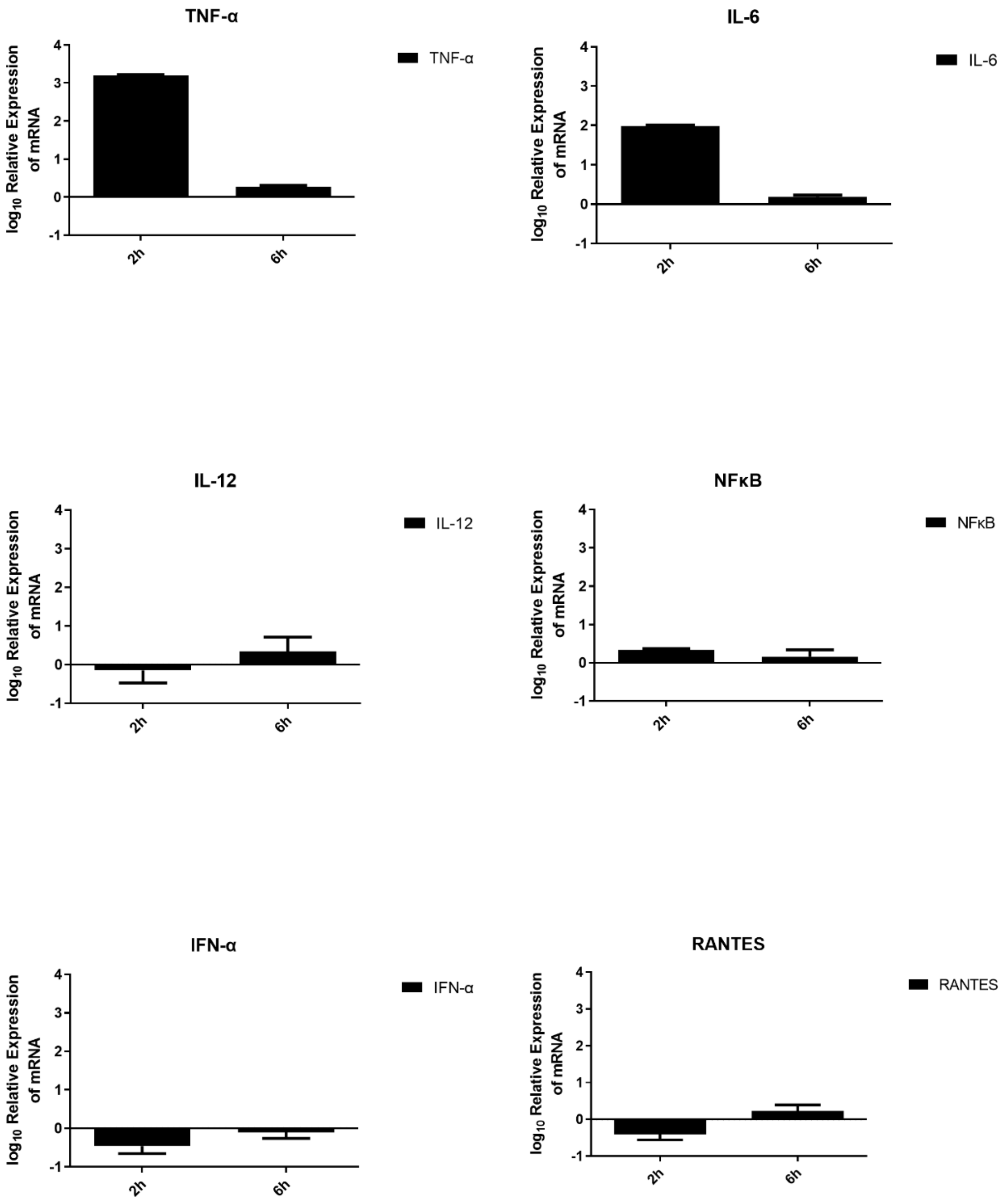

TNF-α
IL-6
IL-12
NFκB
IFN-α
RANTES
log$_{10}$ Relative Expression of mRNA
4
3
2
1
0
-1
2h
6h

H3N2 - ghB

F

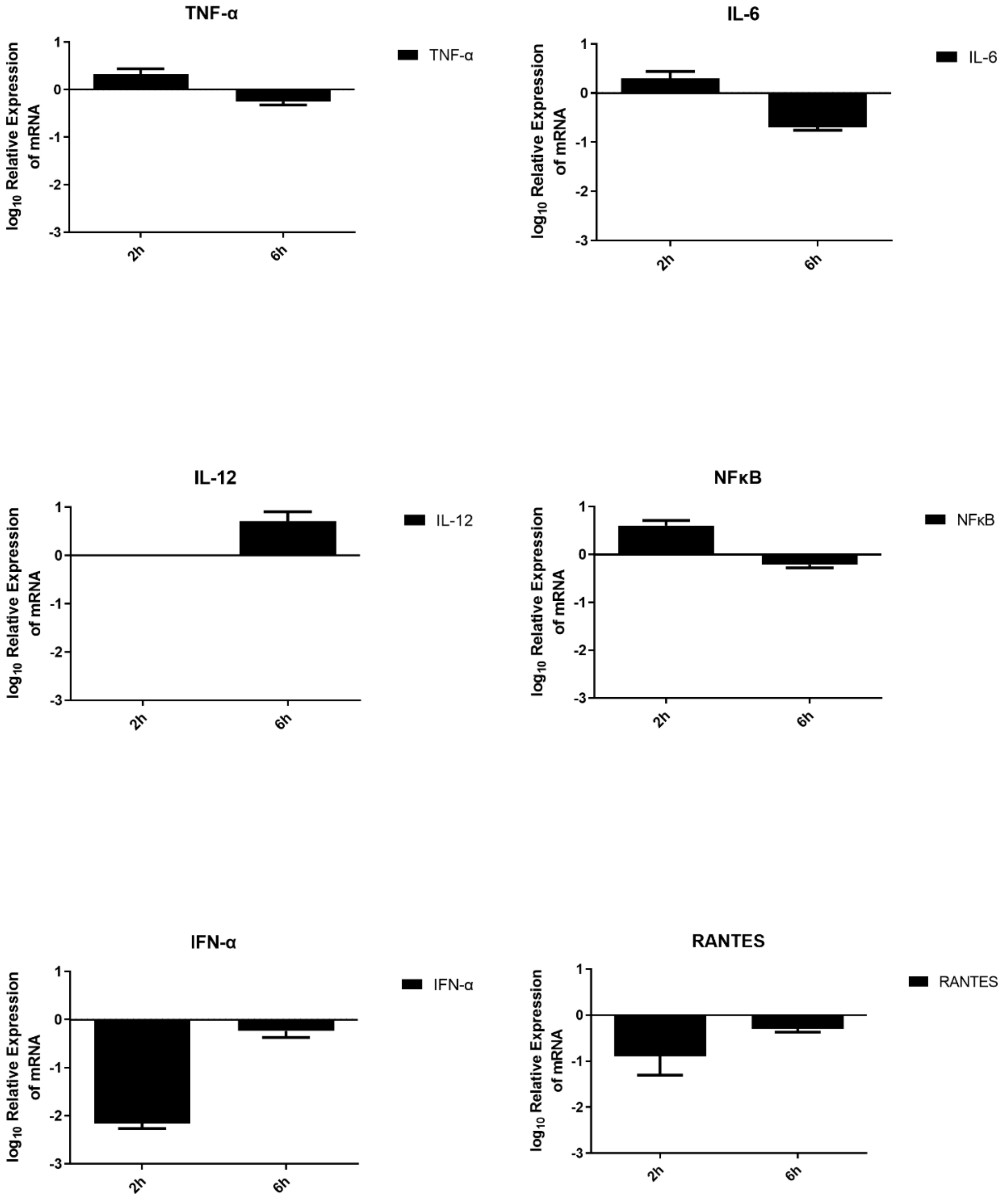
TNF-α
IL-6
IL-12
NFκB
IFN-α
RANTES
log10 Relative Expression of mRNA
1
0
-1
-2
-3
2h
6h

H1N1 - ghC

G

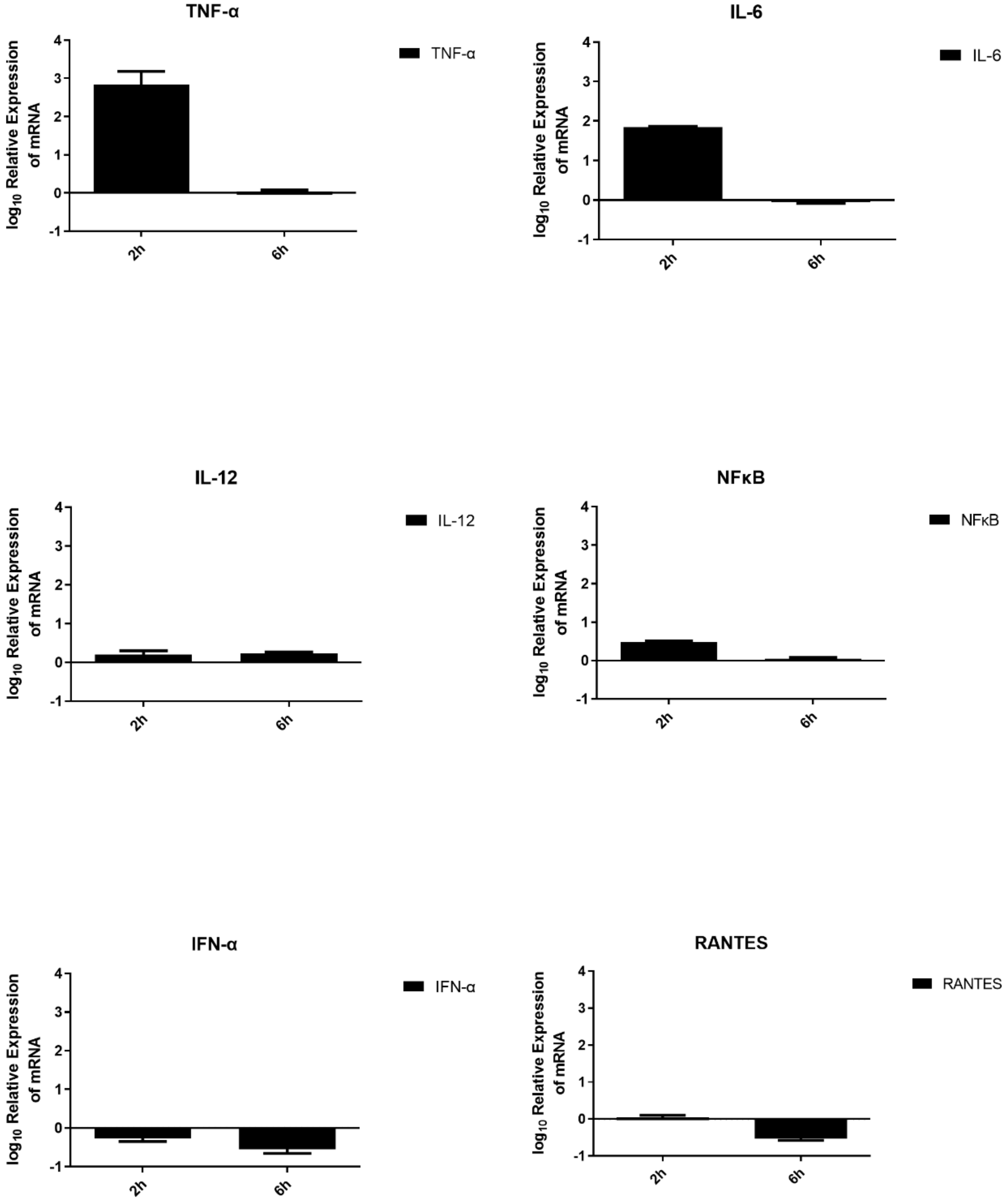
TNF-α
TNF-α
log10 Relative Expression of mRNA
4
3
2
1
0
-1
2h
6h
IL-6
IL-6
IL-12
IL-12
NFκB
NFκB
IFN-α
IFN-α
RANTES
RANTES

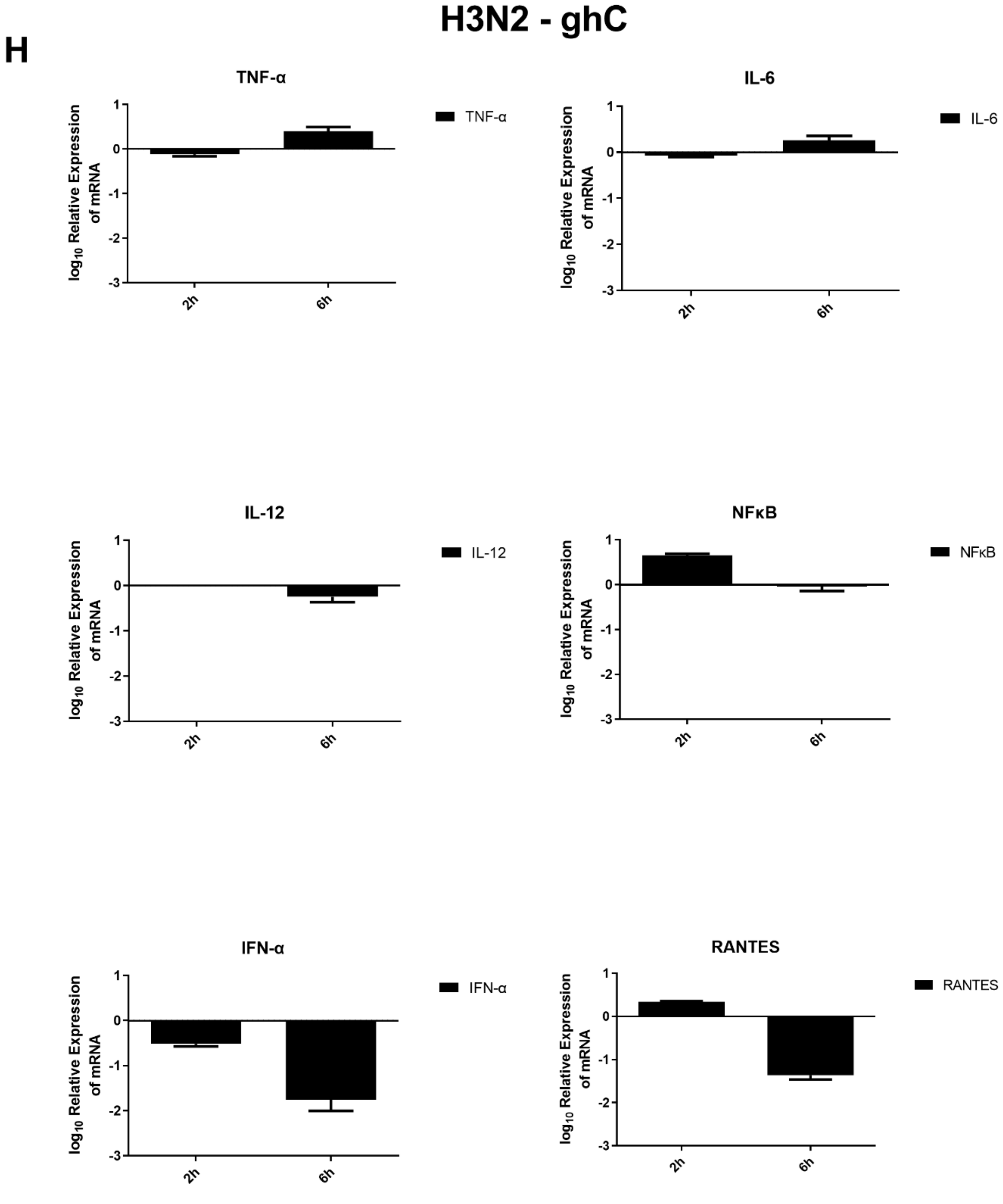

Figure S3**: IAV pre-treatment with human C1q triggers an anti-inflammatory response or pro-inflammatory response in H1N1 or H3N2-infected A549 cells, respectively**

TNF-α, IL-12, IL-6, RANTES, IFN-, and NF-κB mRNA expression levels were evaluated using qRT-PCR using the primers stated in Table 1. As an endogenous control, the data were normalized using 18S rRNA expression. The untreated sample [(C1q: cells + virus only) or (Recombinant MBP fused C1q globular heads: Cells+ virus pre-treated with 20 μg/ml of

MBP)] was used as the calibrator for calculating relative expression (RQ). The RQ value was calculated using the formula: $RQ = 2^{-\Delta\Delta Ct}$.
